# The interaction network of the YidC insertase with the SecYEG translocon, SRP and the SRP receptor FtsY

**DOI:** 10.1038/s41598-017-19019-w

**Published:** 2018-01-12

**Authors:** Narcis-Adrian Petriman, Benjamin Jauß, Antonia Hufnagel, Lisa Franz, Ilie Sachelaru, Friedel Drepper, Bettina Warscheid, Hans-Georg Koch

**Affiliations:** 1 Institute of Biochemistry and Molecular Biology, ZBMZ, Faculty of Medicine, Albert-Ludwigs-University Freiburg, 79104 Freiburg, Germany; 2 Faculty of Biology, Albert-Ludwigs-University Freiburg, 79104 Freiburg, Germany; 3 Institute of Biology II, Biochemistry – Functional Proteomics, Faculty of Biology, Albert-Ludwigs-University Freiburg, 79104 Freiburg, Germany; 4 BIOSS Centre for Biological Signalling Studies, Albert-Ludwigs-University Freiburg, 79104 Freiburg, Germany

## Abstract

YidC/Oxa1/Alb3 are essential proteins that operate independently or cooperatively with the Sec machinery during membrane protein insertion in bacteria, archaea and eukaryotic organelles. Although the interaction between the bacterial SecYEG translocon and YidC has been observed in multiple studies, it is still unknown which domains of YidC are in contact with the SecYEG translocon. By *in vivo* and *in vitro* site-directed and para-formaldehyde cross-linking we identified the auxiliary transmembrane domain 1 of *E. coli* YidC as a major contact site for SecY and SecG. Additional SecY contacts were observed for the tightly packed globular domain and the C1 loop of YidC, which reveals that the hydrophilic cavity of YidC faces the lateral gate of SecY. Surprisingly, YidC-SecYEG contacts were only observed when YidC and SecYEG were present at about stoichiometric concentrations, suggesting that the YidC-SecYEG contact *in vivo* is either very transient or only observed for a very small SecYEG sub-population. This is different for the YidC-SRP and YidC-FtsY interaction, which involves the C1 loop of YidC and is efficiently observed even at sub-stoichiometric concentrations of SRP/FtsY. In summary, our data provide a first detailed view on how YidC interacts with the SecYEG translocon and the SRP-targeting machinery.

## Introduction

The ability to transport proteins from their site of synthesis to their site of function is essential for prokaryotic and eukaryotic cells and involves universally conserved protein machineries that can transport a wide variety of potential substrates^1–4^. An example is the Sec translocon that is found as Sec 61αβγ complex in the endoplasmic reticulum membrane or as SecYEG complex in the bacterial cytoplasmic membrane^5,6^. SecY is composed of 10 transmembrane domains (TMs) and organized as two vestibules with a central constriction, called the pore ring, giving it an overall hour-glass like shape^7^. The constriction is further sealed on the periplasmic side by a small helix, called the plug, which likely helps to maintain the permeability barrier of the protein conducting channel during translocation^8,9^. During protein transport, SecY can open laterally towards the lipid phase by movement of TMs 2B, 3, 7 and 8, which constitute the so called lateral gate^10–12^. SecE in *E. coli* consists of three TMs, which surround the back of SecY and appears to stabilize SecY within the membrane^13,14^. The bacterial SecG contains two TMs and a connecting cytosolic loop and its function is primarily linked to the SecA-dependent translocation of secretory proteins^6,15^.

Although the trimeric SecYEG complex constitutes the minimal membrane embedded unit required for protein transport^16,17^, it associates with multiple partner proteins. This includes SecA, the membrane-associated receptor for secretory proteins^18–22^, and FtsY, the membrane-associated SRP receptor^23–25^. In addition to these membrane-associated partner proteins, SecYEG also interacts with membrane integral proteins, like YidC, the SecDFYajC complex, PpiD or YfgM^15,26–28^.

**Figure 1:**
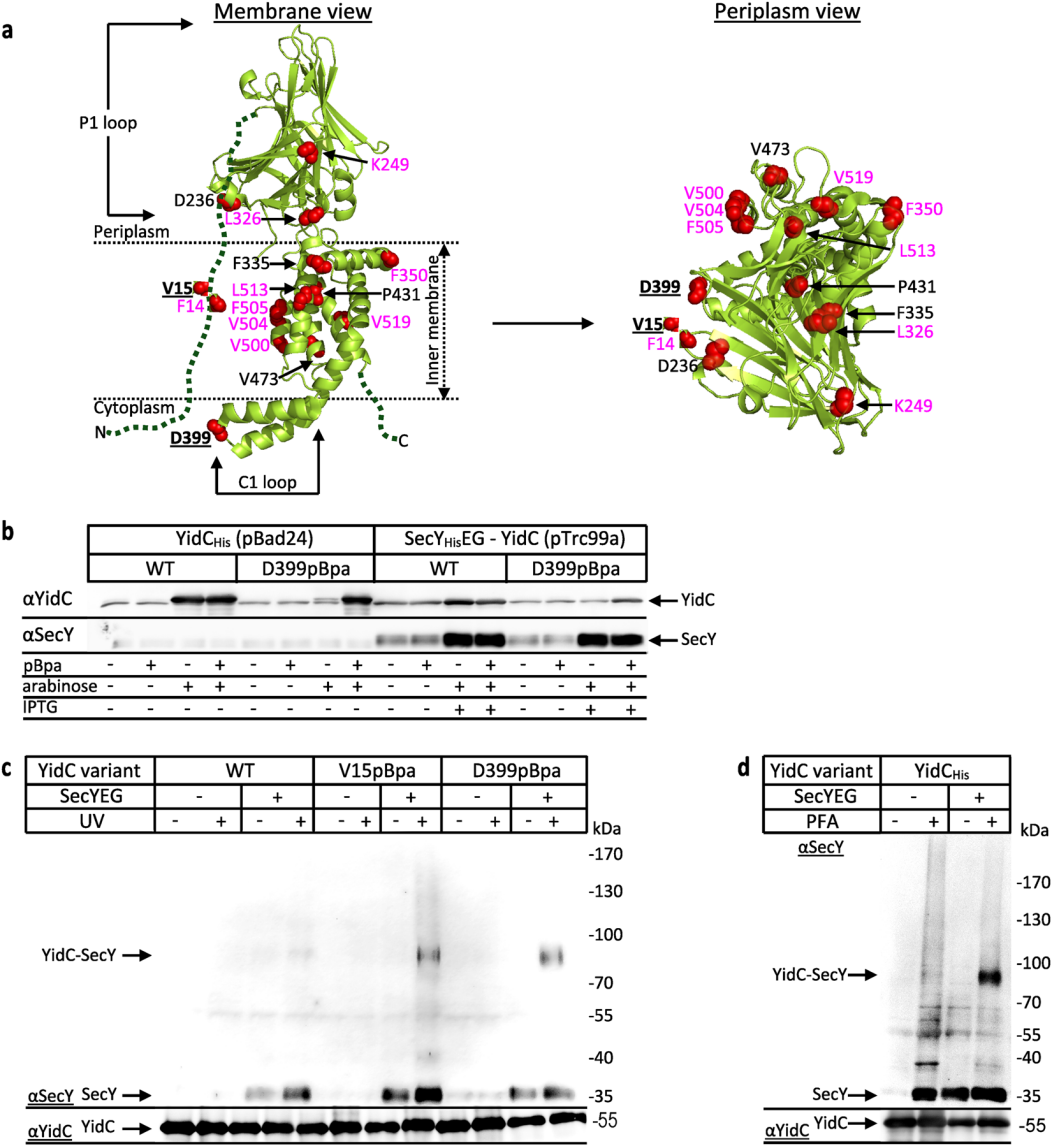
YidC contacts the SecYEG translocon via TM1 and the C1-loop *in vivo*. (**a**) The crystal structure of *E. coli* YidC (PDB accession no.: 3WFV) visualised from the membrane (left) or from the periplasmic side (right). The red spheres indicate the positions for pBpa insertion. Residues which show the strongest contacts to SecY are displayed in bold and underlined. Residues with weaker contacts are shown in black and residues that did not show significant cross-links are shown in magenta. The dashed green lines indicate TM1 and the C-terminus of YidC, which have not been crystallized so far. (**b**) The co-expression system shows balanced expression of YidC and SecYEG as revealed by western blotting. 1 × 10^8^ *E. coli* BL21 cells expressing *yidC* under the arabinose promoter from plasmid pBad24 or co-expressing P_*lac*_*secYEG-yidC* from plasmid pTrc99a were TCA precipitated, separated by SDS-PAGE and after western transfer decorated with the indicated antibodies. WT refers to wild type YidC and D399pBpa to a YidC variant with pBpa inserted at position 399, when pBpa was added to the growth medium. (**c**) *In vivo* photo-cross-linking performed with BL21 *E coli* cells expressing either *yidC* alone (-SecYEG) or co-expressing *yidC* and *secYEG* (+SecYEG). Either wild type YidC (WT) or YidC variants containing pBpa at position V15 or D399 were analysed. After UV-exposure, samples were purified via metal affinity chromatography using an N-terminal His-tag on YidC. A sample without UV-exposure served as a control. Samples were decorated with antibodies against SecY or YidC as indicated. The 95 kDa YidC-SecY cross-link is indicated. (**d**) *In vivo* para-formaldehyde (PFA) cross-linking with BL21 cells expressing either only YidC from plasmid pBad24 or YidC together with SecYEG from plasmid pTrc99a. All samples were treated as described in experimental procedures and YidC was further purified from the membrane fraction via its N-terminal His-tag and analyzed by western blotting using α-SecY antibodies for detecting SecY-YidC cross-links. α-YidC antibodies determined comparable amounts of YidC in all samples. Uncropped images are displayed in Supplementary Figure S4.

YidC contacts the four TMs of the lateral gate of SecY but also penetrates into the SecY channel interior where it interacts with pore ring residues^29,30^. The orientation of YidC at the lateral gate probably allows YidC to assist the release of substrates from the SecY channel into the lipid phase^31,32^. This involves conformational changes at the SecY-YidC interface, including the retraction of YidC from the SecY channel and a reorientation of YidC at the lateral gate^29,30^. YidC consists of a conserved five TM core, which forms a hydrophilic groove that is accessible from both the cytosol and the lipid phase^4,33,34^ (Fig. 1a). In *E. coli*, the five TM core is N-terminally preceded by an additional TM that is connected to TM2 via a large periplasmic domain (the P1 loop) of about 320 amino acids. Two short helices, the C1 loop, connect TM2 and TM3 and are required for the *in vivo* function of YidC^35^. The positively charged C2 loop, connecting TM4 and TM5 constitutes together with the C-terminus of YidC a composite ribosome binding site^35^. YidC is about five-times more abundant than SecYEG^1^ and functions both in complex with SecYEG^27^ but also as SecYEG-independent insertase for some membrane proteins^36–38^.

The contact between YidC and SecYEG is modulated by the SecDFYajC-complex^39,40^, although the SecY-YidC contact is maintained even in the absence of SecDF^29,30^. SecD and SecF are multi-spanning membrane proteins with large periplasmic loops^41^, while YajC is a small, single spanning membrane protein^42^. The SecDFYajC complex likely functions as proton-motive-force powered chaperone complex that aids ATP-independent steps during protein translocation^41^.

The available data indicate the presence of multiple SecYEG translocon assemblies within the membrane, which likely exist in a dynamic equilibrium^4^. The SecYEG-SecDFYajC-YidC complex is the most complex system and referred to as the holo-translocon (HTL)^43,44^. For some substrates it was shown that they are more efficiently transported by the HTL than by SecYEG alone^44^. However, considering the low abundance of SecDF in *E. coli* (approx. 50 copies versus approx. 500 SecYEG copies^1^), only a small number of HTLs can exist in *E. coli*, while there would be enough YidC (2500 copies) to attach to the majority of SecYEG complexes.

Our current understanding of how these accessory proteins connect to the SecYEG core is limited by the lack of high-resolution structures. A low resolution structure of the HTL points to the presence of a lipid-filled cavity at the SecYEG-YidC interface^44^, which could provide a shielded environment for membrane protein insertion and folding. However, it is still unknown which regions of YidC are in contact with SecYEG. Here, we mapped the YidC-SecYEG interface in *E. coli* and identified TM1 and the cytosolic C1 loop of YidC as major contact sites for the SecYEG translocon.

## Results

### The first transmembrane domain and the cytosolic loop of YidC constitute the primary contact site for SecY

By using site-directed cross-linking the SecY positions which are in contact with YidC have been mapped^29,30^. However, using the same approach and inserting the UV-sensitive amino acid derivative para-benzoyl-L-phenylalanine (pBpa)^45^ into different positions within YidC failed to identify by immune detection YidC residues that contact SecY. Only by combining cross-linking with mass spectrometry (MS), the C-terminus of YidC (position YidC540) was found to be in contact with SecY^29^. This was verified in the current study by using the amine-specific cross-linker Disuccinimidyl suberate (DSS) in combination with MS. The identification of the cross-linked peptides revealed that the C-terminus of YidC (residue K545) cross-links to the N-terminus of SecY (position A2) (Table 1). An additional cross-link to the N-terminus of SecY (position K3) was found from the cytosolic loop C1 of YidC (residue 413) (Table 1). These data demonstrated that the C-terminus of YidC and the cytosolic loop C1 (Fig. 1a) are in contact with the N-terminus of SecY. The data further showed that the N-terminal methionine residue of SecY is obviously cleaved, resulting in a free amino group of the succeeding alanine residue.

**Table 1:** The N-terminus of SecY contacts the C-terminus and the cytosolic loop C1 of YidC.

cross-link	cross-linked SecY peptide	cross-linked YidC peptide	calculated mass (Da)	mass deviation (ppm)	E-value
SecY(2)-YidC(545)	_2_AKQPGLDFQSAK_13_	_539_GLHSREKK_546_	2380.2862	2.5	0.0012
SecY(3)-YidC(413)	_2_AKQPGLDFQSAK_13_	_402_QRISQEMMALYKAEK_416_	3251.6681	0.6	0.0247

*E. coli* BL21 cells carrying pTrc99a-SecY_His_EG was used for SecY_His_EG purification via metal affinity chromatography. The amine-specific cross-linker DSS was either added before SecY_His_EG elution (on-column cross-linking) or after elution with elution buffer (solution cross-linking). Subsequently, samples were subjected to HPLC-MS. Cross-linked amino acids in the respective peptides are underlined and numbers in subscript indicate the position of the identified peptide within the protein. Identification of cross-linked peptides was performed essentially as described^77^ using the program pLink®^79^ for searching inter- or intra-molecular cross-links generated by amine-specific cross-linker DSS between peptides from SecY and YidC. The resulting set of peptide spectrum matches at a false discovery rate <0.05 was further filtered requiring a minimum E-value of 0.05 and identification of a pair of cross-linked residues in at least two independent experiments.

Attempts to verify these pBpa or DSS cross-links by immune detection were unsuccessful, suggesting that the experimental conditions allowed only for low-abundant YidC-SecYEG cross-links. This could reflect either a certain flexibility of YidC on the SecY surface, or alternatively, that the stoichiometry between both proteins prevented a more efficient cross-linking. In *E. coli*, YidC is about 5 times more abundant than the SecYEG complex^1^, but performing pBpa or DSS cross-linking with a plasmid-encoded SecYEG results in a surplus of SecYEG over YidC. On the contrary, using plasmid-encoded pBpa-containing YidC further increases the excess of YidC over SecYEG, resulting in only a small number of possible YidC-SecYEG complexes. To overcome this, a co-expression system was established that allowed simultaneous expression of YidC and SecYEG. This system was analyzed for both wild type YidC and for a YidC variant that contained pBpa at position 399 of the C1 loop. The amount of YidC in the co-expression system was lower than in the previously used single YidC expression system (pBad24-YidC), and required for YidC(D399pBpa) the addition of pBpa to the medium for efficient expression (Fig. 1b, compare lanes 7 and 8; and lanes 15 and 16). Without pBpa addition, translation stops at the amber stop codon and only a truncated YidC is synthesized. Importantly, the co-expression system allowed for a significantly increased and IPTG-dependent SecYEG production, compared to the wild type SecYEG levels present in the single expression system (Fig. 1b).

The consequences of simultaneous co-expression of YidC and SecYEG on the *in vivo* cross-linking were analyzed for two YidC variants, which contained pBpa either at position 399 or at position 15 within TM1 of YidC (Fig. 1a,c). TM1 was selected because it is unique to enterobacteria like *E. coli* and not part of the conserved YidC/Oxa core^46^. After UV-exposure of *E. coli* cells, the cross-linked material was purified via an N-terminal His-tag on YidC and probed with α-SecY antibodies. UV-dependent cross-linking products at approx. 95 kDa were detected for both YidC(V15pBpa) and YidC(D399pBpa), but only when SecYEG was co-expressed (Fig. 1c, upper panel). The observed 95 kDa band is in agreement with the previously observed SecY-YidC cross-linking product that was obtained when pBpa was inserted into SecY and which was verified by MS as SecY-YidC complex^29^. The same pBpa-containing YidC variants did not show detectable cross-linking products when expressed without SecYEG co-expression, *i.e*. at the endogenous wild type SecYEG levels (Fig. 1c). This was not related to significant differences in the amount of YidC (Fig. 1c, lower panel), but rather indicates that efficient SecYEG-YidC complex formation as visualized by cross-linking is only observed when SecYEG and YidC are present at about equal concentrations.

An intrinsic limitation of the position-dependent cross-linking is that even subtle changes in pBpa orientation can affect cross-linking. To overcome this limitation and to validate that YidC-SecY cross-links indeed require stoichiometric concentrations of both partner proteins, the amine-group specific, zero-length cross-linker para-formaldehyde (PFA) was used^47^. When cells expressing either YidC or SecYEG-YidC were treated with PFA *in vivo* and YidC was purified via its His-tag, the 95 kDa cross-link product was clearly detected by α-SecY antibodies in the co-expression system, but not when only YidC was expressed (Fig. 1d). This further verifies that about stoichiometric YidC/SecYEG amounts are required for efficient cross-linking. The 95 kDa band was not specifically recognized by the α-YidC antibodies (Supplementary Figure 4), because all YidC cross-linking products were purified via the His-tag purification, rendering a specific detection of a single cross-link species by the α-YidC antibodies unnecessary.

Incidentally, even though no SecY-YidC cross-link was observed without SecYEG co-expression, significant co-purification of native SecY with His-tagged YidC was noticed when PFA was added (Fig. 1d, compare SecY levels). This points to a PFA-stabilized SecYEG-YidC complex, in which SecY and YidC are not in direct contact but rather via accessory subunits of either SecYEG or YidC. In summary, the co-expression system revealed that YidC contacts SecY via TM1 and the C1 loop, which is also in agreement with the DSS cross-linking (Table 1).

**Figure 2:**
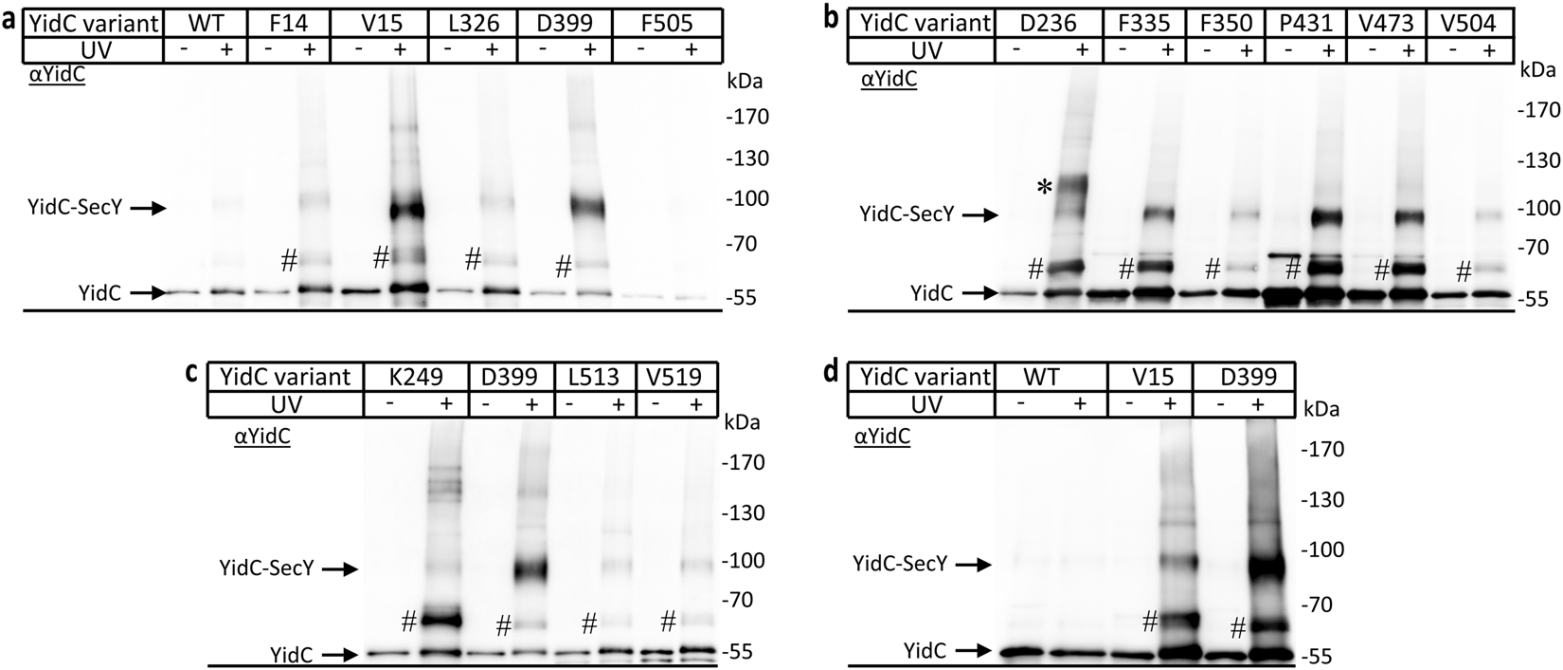
YidC makes multiple contacts to SecY through its transmembrane and periplasmic domains. *In vivo* photo cross-linking was performed as described in Fig. 1 using the co-expression system with YidC variants containing pBpa at the indicated positions. After UV-exposure, samples were purified via the C-terminal His-tag on SecY and analysed on western blot with α-YidC antibodies (**a**–**c**). The 95 kDa YidC-SecY cross-links are indicated. For position D236, an additional strong band below the 130 kDa was recognized by α-YidC antibodies (*). In addition, α-YidC antibodies recognized a UV-dependent band at approx. 65 kDa (#). (**d**) *In vitro* photo cross-linking was performed using inner membrane vesicles carrying 10 μg of wild type YidC or YidC-pBpa variants co-expressed with SecY_His_EG. Samples were purified by metal affinity chromatography using the His-tag on SecY and subsequently analysed on western blot with α-YidC antibodies. Uncropped images are displayed in Supplementary Figure S4.

The co-expression system was then used for identifying additional YidC-SecY contacts. We selected surface exposed residues within the hydrophobic core and within the periplasmic domain of YidC (Fig. 1a). Compared to the strong cross-links at residues V15 and D399, only weak cross-links were observed for residues F14 within TM1, L326 within P1 and F505 in TM5 (Fig. 2a). However, *in vivo* UV exposure of cells expressing wild-type YidC (*i.e*. without pBpa insertion) also resulted in a very weak 95 kDa cross-linking product (Fig. 2a). This is probably the result of UV-dependent radical formation of aromatic amino acids that favors non-specific protein-protein and protein-nucleic acid cross-links^48,49^. Thus, the weak cross-linking products do not necessarily reflect site-specific contacts. Strong 95 kDa YidC-SecY cross-links were observed for residues F335, P431 and V473 and weaker cross-links for residues D236, F350 and V504 by α-YidC antibodies (Fig. 2b). Additional weak SecY contacts were observed for residue K249 in P1 and residues L513 and V519 within TM6 (Fig. 2c). The strong YidC-SecY cross-links were also detected by α-SecY antibodies (Supplementary Fig. S1), but the quality of western blots using α-SecY antibodies is generally lower, and requires extensive purification of the cross-linking products.

Residue D236 within the P1 loop showed an additional cross-link product below the 130 kDa marker band (Fig. 2b,∗), which is likely identical to the about 130 kDa SecY-YidC cross-link product that was identified by MS when pBpa was incorporated at position 127 within TM3 of SecY^29^. It could correspond to a YidC-(SecY)_2_ complex, however, as the appearance of the 130 kDa cross-linking product appeared to be strain dependent, *i.e*. it is visible in strains C43 and BL21 but not in BL325^29,30^, this was not further analyzed. Finally, for all residues showing a clear YidC-SecY cross-linking product, an additional cross-link migrating below the 70 kDa marker band was recognized by α-YidC antibodies (Fig. 2#; see below for its characterization).

In summary, these data revealed that YidC makes multiple contacts to SecY via TM1 (V15), the periplasmic domain (F335), the C1 loop (D399), TM3 (P431) and TM4 (V473) (Fig. 1a). Additional weak contacts were observed for F14 (TM1), D236/L326/F350 (P1), V504 (TM5) and L513/V519 (TM6). However the intensity of these weaker cross-links was identical or only slightly stronger than those observed for wild type YidC and thus do not necessarily reflect position-dependent cross-links. Making quantitative assessments based on non-equilibrium methods like cross-linking is difficult, but the intensity of the YidC-SecY cross-linking product from the different pBpa positions suggested that TM1 and the C1 loop are major contact sites for SecY. This was further verified by *in vitro* cross-linking using purified inner membrane vesicles (INV) of *E. coli* cells co-expressing SecYEG and YidC(pBpa), which also revealed strong contacts between SecY and YidC residues V15 and D399 (Fig. 2d).

### Position dependent contacts of YidC with SecE and SecG

**Figure 3:**
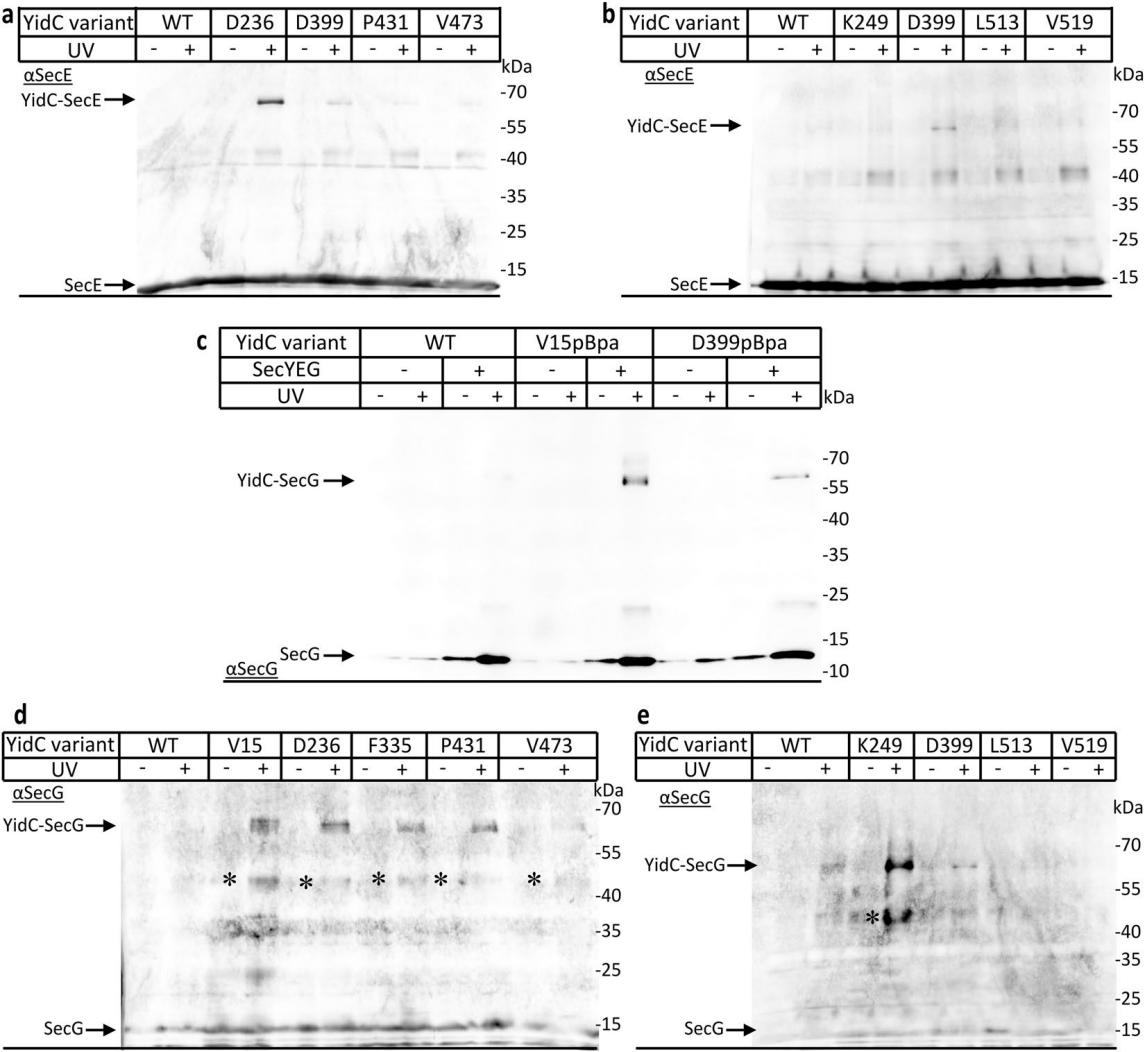
YidC contacts the SecY associated proteins SecE and SecG. The cross-linked and purified material prepared for Figs 1c and 2a,c was decorated with antibodies against SecE (**a**,**b**) or SecG (**c**–**e**). (**c**) The samples were analysed with antibodies against SecG both at the endogenous SecYEG levels (-SecYEG) and in the co-expression system (+SecYEG). The cross-link products are indicated. The UV dependent 45 kDa bands (*) in Fig. 2d–e likely represent a proteolytic cleavage product of the YidC-SecG cross-links. Uncropped images are displayed in Supplementary Figure S4.

The α-YidC antibodies recognized not only the 95 kDa band but also a UV-dependent band at approx. 65 kDa (*c.f*. Fig. 2,#) and the intensity of this band largely coincided with the intensity of the 95 kDa SecY-YidC cross-link. We therefore reasoned that the 65 kDa band could reflect YidC cross-links to either SecE or SecG, which both have a MW of about 13 kDa^13^. α-SecE antibodies clearly recognized the 65 kDa band in the YidC(D236pBa) variant and weakly also for YidC(D399pBpa) (Fig. 3a). pBpa insertions at the other residues (F14, V15, L326, F335, F350, P431, V473,V504) did not show the 65 kDa band or only at the detection limit of the α-SecE antibodies (Fig. 3 and Supplementary Figure S2). These data demonstrate that the YidC-SecE contact primarily involves the periplasmic loop of YidC (D236) and weakly the cytoplasmic region C1, but no intra-membrane residues.

As the 65 kDa band was not recognized by α-SecE antibodies for all YidC(pBpa) variants, we assumed that for some YidC residues, it reflects a YidC-SecG cross-link. α-SecG antibodies recognized the 65 kDa band for V15 and also with lower intensity for D399 in the co-expression system, but not at the endogenous SecYEG levels (Fig. 3c). YidC-SecG cross-links were also observed for residues D236, K249, F335, P431 and (weakly) for V473 (Fig. 3d,e). Residues F350, V504, L513 and V519 gave no cross-linking product (Fig. 3e and Supplementary Figure S2). A UV-dependent band at 45 kDa was recognized by the α-SecG antibody in some samples (Fig. 3,*). This band could reflect a proteolytic fragment of the YidC-SecG cross-link product, but this was not further analyzed in the current study. Thus, YidC contacts SecG via TM1 (V15), the periplasmic loop (D236, F335, K249) and TM4 (P431), while no significant contacts were observed for pBpa insertions in TM5 and TM6.

**Figure 4:**
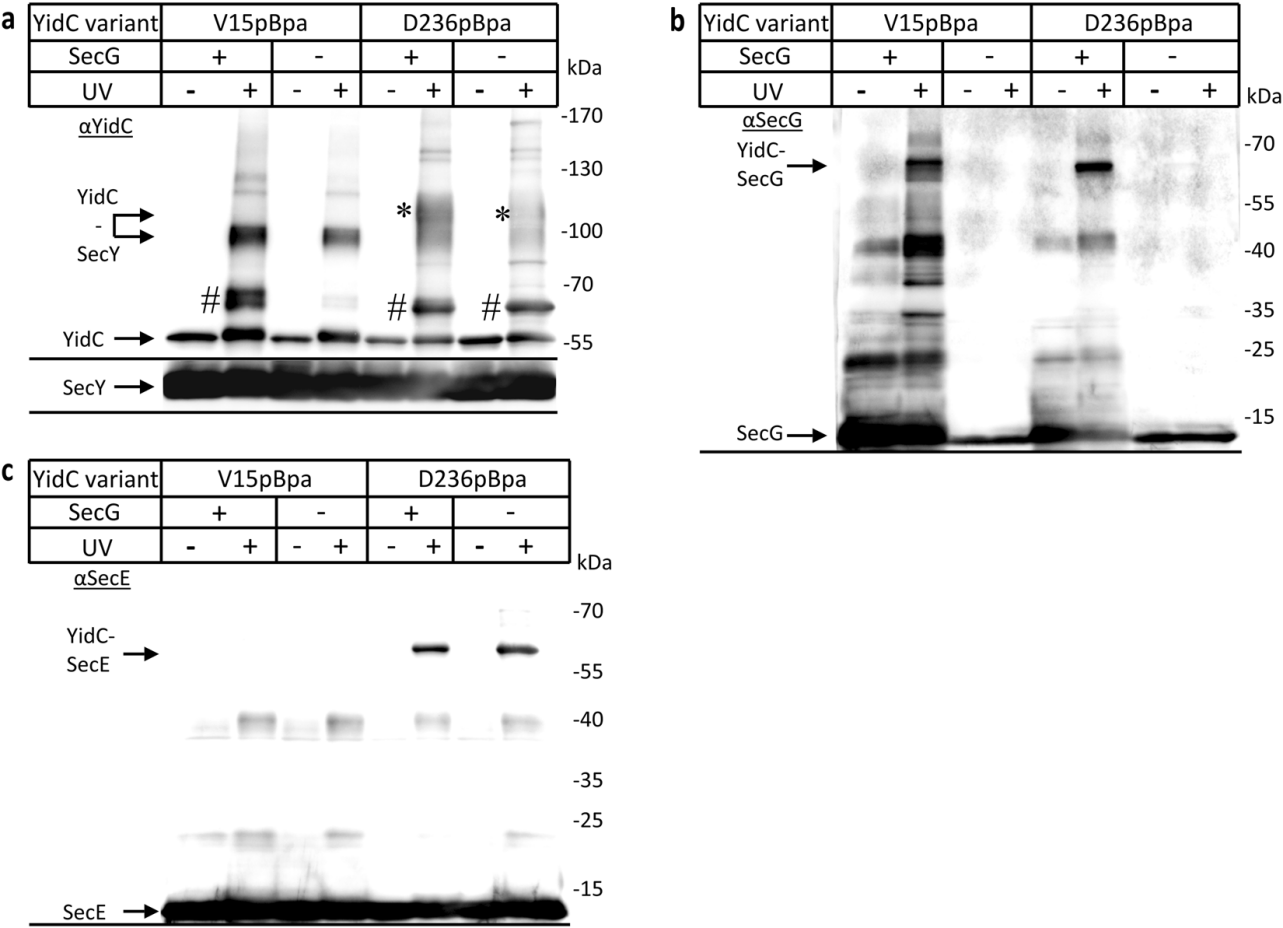
The periplasmic loop of YidC contacts SecE and SecG, while TM1 is only in contact with SecG. *In vivo* photo-cross-linking was performed with a SecY_His_EG-YidC or SecY_His_E-YidC co-expression system with pBpa incorporated either in TM1 (position 15) or within the P1 loop (position 236) of YidC. After purification via the C-terminal His-tag on SecY, the samples were analysed using antibodies against YidC (**a**), SecG (**b**) or SecE (**c**). The 95 kDa YidC-SecY cross-link and the 65 kDa YidC-SecE/G cross-links are indicated (#). For pBpa-insertion at position 236 of YidC, in addition to the 95 kDa YidC-SecY cross-link, a second cross-link product below the 130 kDa marker was observed (*), as already shown in Fig. 2a. Uncropped images are displayed in Supplementary Figure S4.

While SecE is essential for the stability of SecY^14^, SecG can be deleted^50^. This allowed us to further differentiate between YidC-SecE and YidC-SecG cross-links. YidC(pBpa) variants were either co-expressed with SecYEG or with just SecYE. In the absence of stoichiometric SecG amounts, the 95 kDa YidC-SecY cross-link was detected by α-YidC antibodies for YidC(V15pBpa), indicating that TM1 of YidC maintains contact to SecY even in the absence of SecG (Fig. 4a). This was different for YidC(D236pBpa), which showed a significant reduction of both the 95 kDa and the 130 kDa cross-linking products when SecG was not co-expressed (Fig. 4a). Thus, the presence of SecG appears to enhance the interaction of the P1 loop of YidC with SecY. The 65 kDa band that reflects either a YidC-SecE or a YidC-SecG cross-link was largely absent for YidC(V15pBpa), but unchanged for YidC(D236pBpa) when SecG was not co-expressed (Fig. 4a,#).

α-SecG antibodies recognized the 65 kDa cross-link band only when SecG was present in stoichiometric amounts (Fig. 4b). In contrast, α-SecE antibodies recognized the 65 kDa band for position D236 both in the absence and presence of SecG (Fig. 4c), while they failed to detect a band at 65 kDa for position V15 in YidC. These results confirm that the 65 kDa cross-link band corresponds to both YidC-SecE and YidC-SecG complexes and reveal that TM1 of YidC makes exclusive contact to SecG, while the P1 loop is contact with both SecE and SecG.

### The C1 loop is essential for YidC function but not essential for the interaction with SecYEG

**Figure 5:**
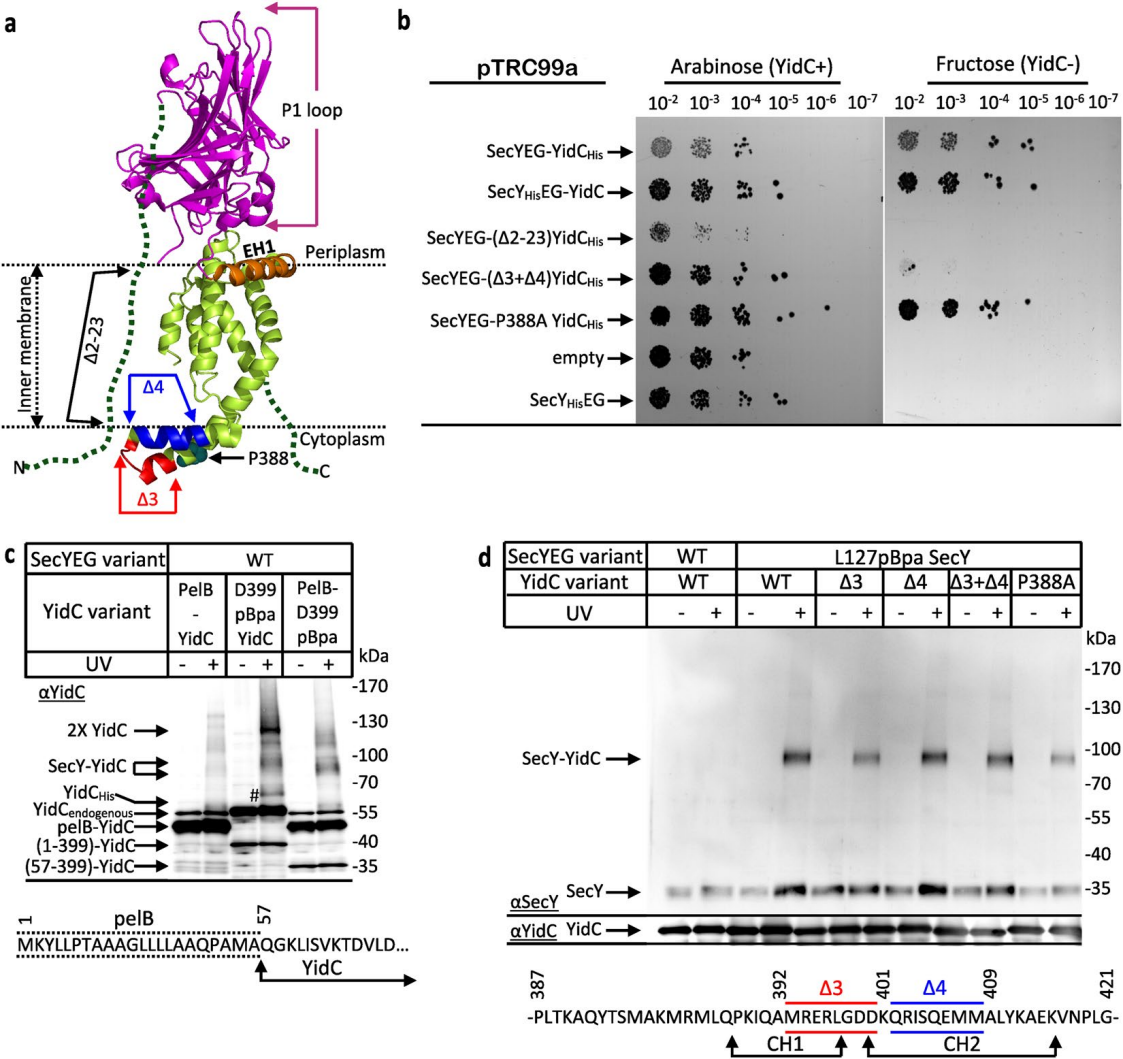
The C1-loop and TM1 form a composite binding site for SecY. (**a**) The positions of the partial YidC deletions used in (**b**) (Δ3 + Δ4 and Δ2-23) and (**d**) (Δ3, Δ4, Δ3 + Δ4) as well as the position of proline 388 which was mutated to alanine (P388A) (**b**,**d**) are indicated on the *E. coli* YidC crystal structure (PDB accession no. 3WFV). In addition, the amphipathic helix EH1 (orange) and the periplasmic loop 1 (magenta) are displayed. The dashed lines indicate TM1 and the C-terminus of YidC for which no structural information is available. (**b**) The conditional YidC depletion strain JS7131 was used to monitor the functionality of different pTrc99a-YidC-SecYEG co-expression systems. The presence of arabinose in the media results in expression of the endogenous YidC via the *araBAD* promoter while the absence of arabinose (replaced by fructose) induces YidC depletion. SecY_His_EG-YidC corresponds to a co-expression system with a C-terminal His-tag on SecY, SecYEG-YidC_His_ to the co-expression system with a N-terminal His-tag on YidC; SecYEG-YidC_His_-(Δ2-23)YidC_His_ to a co-expression system in which YidC lacked TM1 (residues 2–23), SecYEG-(Δ3 + Δ4)YidC_His_ to a co-expression system in which most of the C1 loop was deleted, SecYEG-(P388A)YidC_His_ to a co-expression system in which proline 388 of YidC was replaced by alanine. The empty plasmid (pTrc99a) or pTrc99a expressing just SecY_His_EG served as control. Cells were sequentially diluted in LB medium and spotted onto LB plates with or without arabinose; cell growth was monitored after overnight incubation at 37 °C (upper panel). (**c**) *In vivo* photo-cross-linking was performed in the co-expression system with a YidC variant in which TM1 was replaced by the PelB signal sequence (PelB-YidC). When indicated pBpa was inserted at position 399 of either PelB-YidC (PelB-D399pBpa) or wild type YidC (D399pBpa-YidC). After UV-exposure of 1 × 10^8^ cells, the material was not further purified but directly TCA precipitated and analysed by western blotting using α-YidC antibodies. Indicated are the cross-links (SecY-YidC), the endogenous YidC (YidC_endogenous_), PelB-YidC and the truncated YidC variants produced by incomplete suppression of the amber stop codon at position 399. In addition, a YidC dimer is indicated (2 × YidC). The N-terminal sequence of the PelB-YidC variant is displayed in the lower panel. The 65 kDa cross-link products between YidC and SecE/SecG are indicated (#) (**d**) *In vivo* photo-cross-linking was performed in the co-expression system with the indicated YidC variants. YidC was purified by metal affinity chromatography via an N-terminal His-tag on YidC and further analysed by western blotting with antibodies against SecY and YidC. The sequence information of the deletion mutants used in this experiment is displayed in the lower panel. CH1 and CH2 correspond to the two α-helices of the C1-loop. Uncropped images are displayed in Supplementary Figure S4.

The C1 loop of YidC is in contact with SecY and SecE and we therefore determined whether it is essential for the YidC-SecYEG interaction. The loop consists of two helices (CH_1_ and CH_2_), which are required for the *in vivo* function of YidC^35,51^. YidC loop mutants were generated in which residues 392–401 of CH_1_ (YidCΔ3) or residues 401–409 of CH_2_ (YidCΔ4) were deleted (Fig. 5a,d). In addition, a YidC mutant was generated which lacked residues 392–409 (YidCΔ3 + Δ4) and a mutant in which the conserved proline residue at position 388 was replaced by alanine. The latter is expected to change the orientation of the loop (Fig. 5a). The functionality of these YidC mutants was tested in the conditional YidC-depletion strain JS7131^36^, which contains *yidC* under the control of the arabinose promotor and requires arabinose-supplementation for growth. When this strain was transformed with the empty vector (pTrc99a) or pTrc99a-SecY_His_EG, it grew only on arabinose-supplemented plates (Fig. 5b, 6^th^ and 7^th^ row). In contrast, cells containing the co-expression plasmid pTrc99a-SecYEG-YidC grew both in the presence and absence of arabinose, validating that the co-expression system produced active YidC. Complementation was observed for both the His-tagged and non-His-tagged YidC (Fig. 5b, 1^st^ and 2^nd^ row), demonstrating that the N-terminal His-tag on YidC does not interfere with function. Arabinose-independent growth was also observed for the YidC(P388A) mutant (Fig. 5b, 5^th^ row), indicating that this particular residue is not essential for YidC function. However, JS7131 cells expressing YidC(Δ3 + Δ4) were arabinose dependent, showing that the C1 loop is required for YidC function (Fig. 5b, 4^th^ row), which is in agreement with previous studies^51^. We also constructed a YidC variant lacking TM1 (YidCΔ2-23) and JS7131 expressing this mutant was also strictly arabinose dependent (Fig. 5b, 3^rd^ row), initially suggesting that TM1 is essential for YidC function. However, further proteolysis experiments revealed that YidCΔ2-23, although expressed (Supplementary Figure S3a), is not properly inserted into the *E. coli* membrane and therefore this mutant was not further characterized in the current study. Instead, we generated a YidC variant in which TM1 was replaced by the cleavable PelB signal sequence (Fig. 5c). When co-expressed with SecYEG, this construct complemented the JS7131 strain (Supplementary Figure S3b), confirming that TM1 is not essential under the conditions tested^52^. Immune-detection of these cells revealed expression of pelB-YidC and signal sequence cleavage, as indicated by the faster migration on SDS-PAGE (Fig. 5c). We then tested whether the YidC-SecY contact in the co-expression system was influenced by the absence of TM1. pBpa was inserted at position 399 of either YidC or pelB-YidC and cells were UV-exposed. Detection of the YidC-SecY cross-links was then performed without further purification, *i.e*. in crude cell extracts. The YidC-SecY cross-link was observed for pelB-YidC(D399pBpa), migrating slightly below the 95 kDa cross-link observed for YidC(D399 pBpa) (Fig. 5c). However, the 65 kDa SecE/SecG cross-link products (Fig. 5c,#) were not clearly detected with pelB-YidC. These data demonstrate that the YidC-SecY contact is maintained even when TM1 of YidC is missing, while contact to SecE and SecG is reduced.

The impact of the C1-mutants on the SecYEG-YidC interaction was also monitored in the co-expression system. Here, pBpa was inserted into position L127 of SecY, which is located in TM3 of SecY, one of the lateral gate helices that have been found to contact YidC^29^. For all C1 loop mutations, the SecY-YidC cross-linking band at 95 kDa was still observed (Fig. 5d), demonstrating that the YidC-SecYEG contact via the lateral gate of SecY is maintained even when the C1 loop of YidC is truncated. In summary, these data indicate that TM1 and the C1-loop constitute a composite binding site for SecY and that either one is sufficient to maintain the YidC-SecY interaction.

### The cytosolic loop of YidC also binds SRP and the SRP receptor

The C1 loop seems to be dispensable for the insertion of some, but not all YidC substrates. As some substrates are targeted to YidC by the SRP system^35,53^, we analysed whether the C1 loop is involved in binding SRP or the SRP receptor FtsY. These cross-linking experiments were performed in the single expression system, *i.e*. without simultaneous SecYEG co-expression. The rationale here was to monitor only direct contacts to YidC and not those that were mediated by initial binding of SRP/FtsY to SecYEG in SecYEG-YidC complexes.

**Figure 6:**
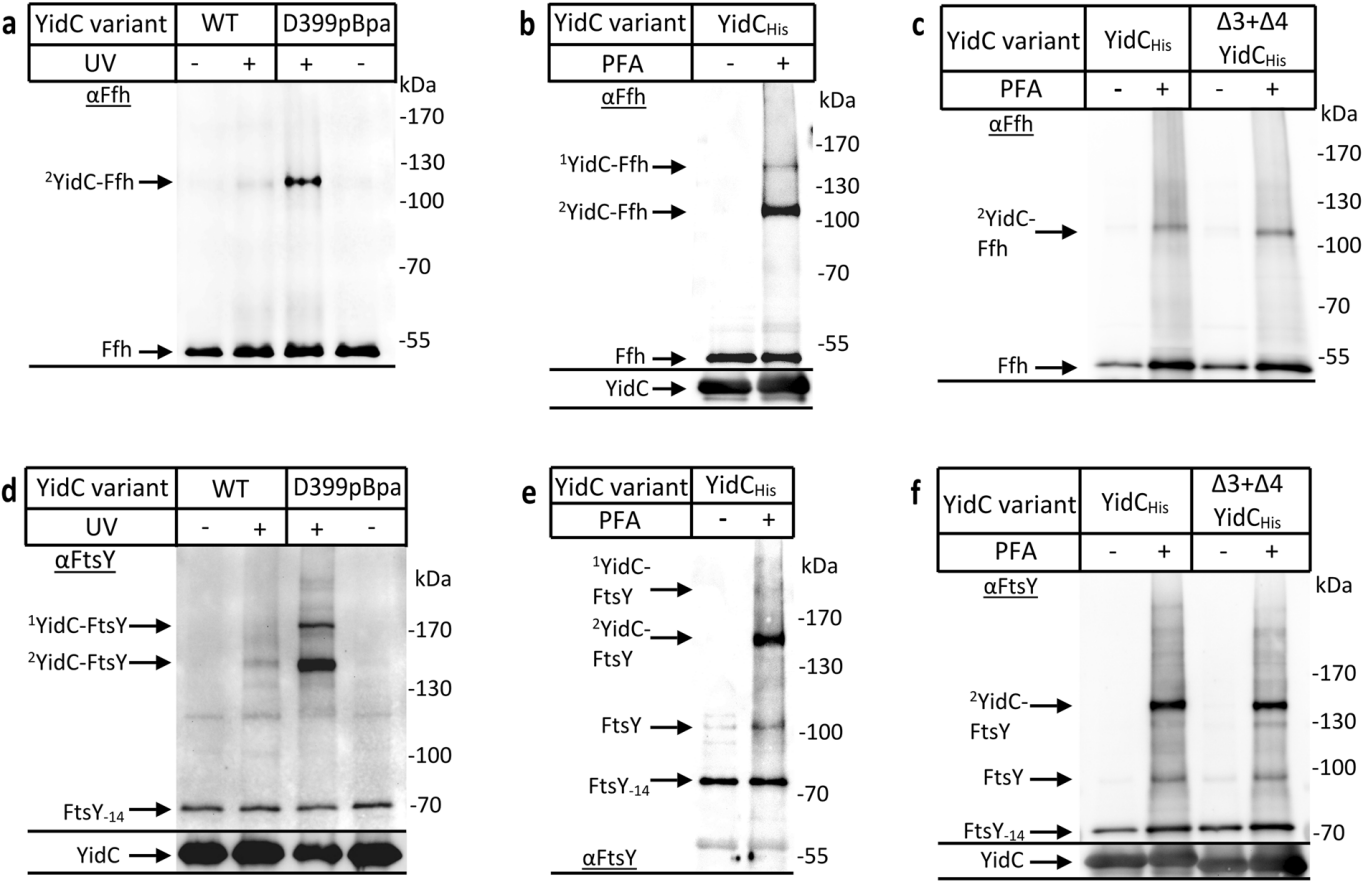
The cytosolic loop 2 of YidC constitutes a binding site for SRP and its receptor FtsY. (**a**) *In vivo* photo-cross-linking was performed in the single-expression system using BL21 cells expressing *yidC* with pBpa at position 399. Subsequently YidC was purified via metal affinity chromatography and analysed on western blot with antibodies directed against Ffh, the protein subunit of the *E. coli* SRP. The 110 kDa YidC-Ffh cross-link is indicated. Note, a very weak, apparently also UV-dependent band was recognized by α-Ffh antibodies in the wild type. (**b**) BL21 cells expressing YidC were treated *in vivo* with p-formaldehyde (PFA) or buffer as a control. Subsequently, YidC was purified together with its cross-linking partners by metal affinity chromatography and analysed on western blot with α-Ffh antibodies. The ^1^YidC-Ffh species likely corresponds to a YidC-Ffh-4.5SRNA cross-link product and the ^2^YidC-Ffh species to the YidC-Ffh cross-link. (**c**) As in (b), but with cells expressing either wild type YidC or the YidC(Δ3 + Δ4) variant, which lacks most of the C1-loop. (**d**) The same material shown in (a) was decorated with α-FtsY antibodies, which revealed two cross-link products migrating at about 150 kDa and 180 kDa. FtsY-14 corresponds to the proteolytic cleavage product of FtsY, which is frequently observed. (**e**,**f**) The same material as in (**b**,**c**) was decorated with α-FtsY antibodies. Uncropped images are displayed in Supplementary Figure S4.

The cross-linked material from cells expressing YidC(D399pBpa) was first probed with antibodies against Ffh (48 kDa), the protein component of the bacterial SRP. A UV-dependent band was recognized by the α-Ffh antibody at approx. 110 kDa, in line with the predicted mass of a YidC-Ffh complex (Fig. 6a) and demonstrating that SRP interacts with the C1 loop of YidC. Whether the C1 loop was essential for the contact between YidC and SRP was analysed by using PFA cross-linking *in vivo*. After PFA cross-linking, YidC was purified and the material analysed by immune detection. α-Ffh antibodies recognized two PFA-dependent bands, the 110 kDa band that was also seen by pBpa-cross-linking and a second band at approx. 150 kDa (Fig. 6b). Different to pBpa, PFA can induce cross-links between multiple components and therefore the 150 kDa band likely reflects an Ffh-4.5 S RNA-YidC complex. Together with Ffh, the 35 kDa 4.5 S RNA forms the *E. coli* SRP^54^. The additional band would also be consistent with a SecY-YidC-Ffh cross-link. However, we consider this less likely, because the detection of a SecY-YidC complex by PFA cross-linking required the SecYEG-YidC co-expression and antibodies against SecY failed to detect a 150 kDa band (*c.f*. Fig. 1d), The PFA approach was then tested for YidC(Δ3 + Δ4), but we observed no significant reduction in the Ffh cross-linking efficiency compared to wild type YidC (Fig. 6c).

The same strategy was also used for monitoring possible interaction between the C1 loop and FtsY. α-FtsY antibodies recognized two UV dependent bands at approx. 150 kDa and 180 kDa for YidC(D399pBpa) (Fig. 6d). FtsY exists in two isoforms migrating at 100 kDa and 70 kDa, respectively^55,56^ and the two cross-link species probably reflect contacts of YidC with both isoforms, although only the proteolytically processed 70 kDa FtsY-14 isoform co-purified with YidC (Fig. 6d). The 180 kDa cross-link species would also fit in size with a SecY-YidC-FtsY complex, but SecY antibodies failed to recognize a 180 kDa complex and pBpa within YidC can only cross-link to one partner protein. PFA-cross-linking confirmed the presence of a strong 150 kDa and a weak 180 kDa YidC-FtsY complex (Fig. 6e), but as for Ffh this contact was maintained even in the YidC(Δ3 + Δ4) mutant (Fig. 6f). Thus, although the C1 loop is important for YidC interactions and essential for function, it is not essential for the interactions with SecYEG, FtsY, or SRP.

**Figure 7:**
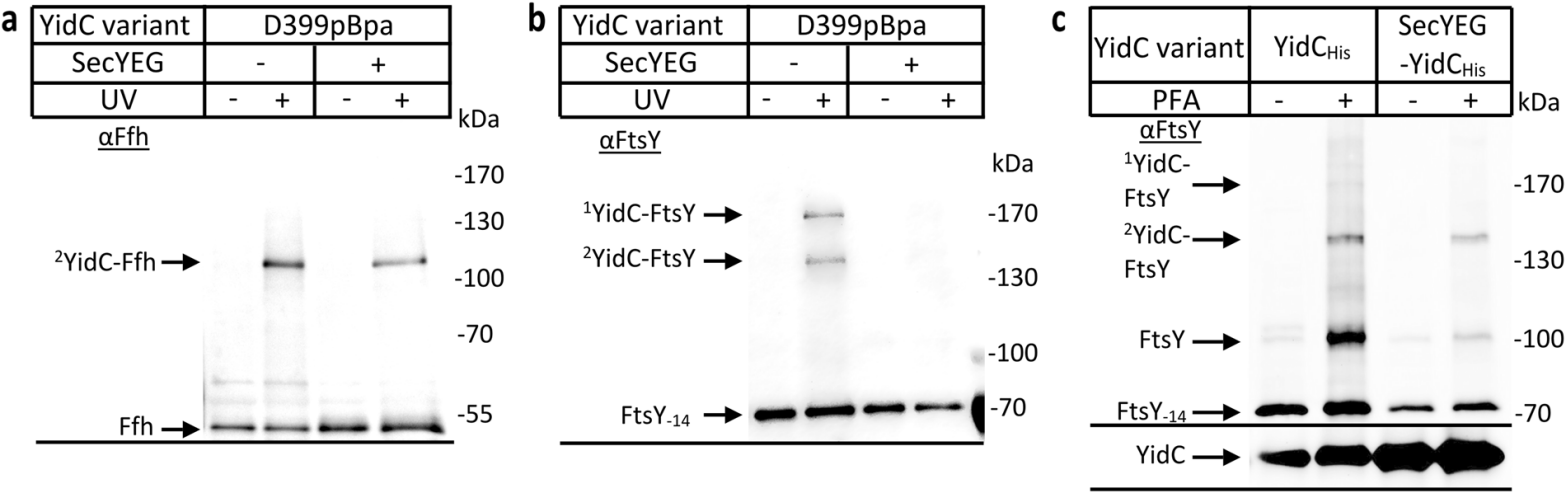
The presence of SecYEG prevents binding of FtsY to the C1 loop of YidC. *In vivo* photo cross-linking was performed with BL21 cells expressing either just YidC or together with SecYEG. In both cases pBpa was present at position 399 of YidC. YidC was purified via its His-tag and analysed using antibodies against Ffh (**a**) or FtsY (**b**). Note that the YidC amounts which are present in (**a**) and (**b**) are displayed in Fig. 1c (α-YidC -bottom). (**c**) *In vivo* p-formaldehyde cross-linking was performed with BL21 cells expressing YidC only or together with SecYEG. The cross-linked material was analysed with α-FtsY (top) and α-YidC (bottom) antibodies. Uncropped images are displayed in Supplementary Figure S4.

In contrast to the SecYEG-YidC interaction, which required their simultaneous co-expression to be detected, the interaction between Ffh/FtsY and YidC was detectable at the endogenous Ffh/FtsY levels. As Ffh is considered to exist in only about 200–300 copies per cell^1^, a significant portion of the cellular Ffh is obviously in direct contact with YidC. This interaction was still detectable when SecYEG was co-expressed, but became slightly weaker (Fig. 7a). Although direct contacts between SecYEG and Ffh have not been analysed so far, the likely explanation for the reduction is that a portion of Ffh is now recruited to SecYEG.

Different to Ffh, FtsY is a well-studied partner protein of SecYEG^23,25,57^ and FtsY contact to position D399 of YidC was lost when SecYEG was co-expressed (Fig. 7b). Thus, in the presence of increased SecYEG concentrations, the majority of FtsY is obviously recruited to SecYEG. Alternatively, as the C1 loop also provides a binding site for SecYEG, the C1 loop could be re-oriented or shielded in the YidC-SecYEG complex, preventing cross-links to FtsY from position D399 of YidC. This was further analysed by PFA cross-linking, which revealed a significant reduction of the YidC-FtsY interaction when SecYEG was co-expressed (Fig. 7c). This position-independent cross-linking experiment supports the conclusion that the majority of FtsY is recruited to SecYEG in the co-expression system, suggesting that FtsY has a higher affinity for SecY than for YidC.

## Discussion

Our study has revealed two unexpected aspects of the SecYEG-YidC assembly: (1) The non-conserved TM1 of *E. coli* YidC provides a major contact site for SecY and SecG and (2) efficient contacts between YidC and SecYEG as visualized by cross-linking are only observed when SecYEG is overexpressed. In addition, our data demonstrate that the C1 loop is an important hotspot for the interaction with SecYEG, SRP and FtsY.

YidC-like proteins facilitate protein transport in all three domains of life^36,58–60^ and they work autonomously for some substrates^36^, but also cooperate with the SecYEG translocon during membrane protein insertion^27,43^. In the resting SecYEG channel, YidC contacts the lateral gate and also penetrates deeply into the channel. Upon substrate insertion, YidC is expelled from the channel, but maintains contact to the lateral gate^29,30^, likely providing a sheltered substrate binding surface for TMs exiting SecY^61,62^. Our understanding on how YidC interacts with the SecYEG translocon is so far limited because the regions within YidC that contact the Sec translocon have not been identified, with the exception of a SecF binding site within the P1 loop^40^. Based on a genetic screen, residue G355 at the interface of P1 and TM2 and M471 within TM4 were predicted to contact SecY^63^. This is in line with the assumption that the conserved hydrophilic substrate binding groove of YidC probably faces the SecY lateral gate^34^. Our data now show that TM1 of YidC constitutes together with the C1 loop the major contact site for the SecYEG translocon. The exact localization of TM1 in *E. coli* YidC has not been resolved yet, but cysteine scanning mutagenesis has revealed that TM1 is involved in substrate binding, together with TMs 3–5^64,65^. This indicates that TM1 is located close to the YidC core, although the connecting P1 loop likely provides some flexibility. TM1 is the most likely candidate to enter the interior of the SecY channel, because the other YidC TMs are tightly packed together at the periplasmic leaflet of the membrane^62^ and movements of these TMs would probably disrupt the hydrophilic substrate binding groove. The proposed flexibility of TM1 would also explain why cross-links to both SecY and SecG were observed. It is important to note that the *in vivo* cross-linking approach is not synchronized and thus monitors the YidC-SecY contact at different interaction states. This also explains why multiple surface exposed residues of YidC were found to contact SecY. Previous data had already demonstrated that YidC undergoes significant rearrangements at the SecY surface upon substrate insertion^29,30^ and this is likely represented by the number of cross-linking residues. As cross-linking reports on the proximity between two molecules, the weaker cross-link species could also result from a random collision between SecY and YidC. Still, the major contacts at TM1 and C1 were also confirmed by *in vitro* cross-linking using INVs, representing a resting state. TM1 and the P1 loop of *E. coli* YidC comprise 356 amino acids, yet they are largely dispensable for YidC function. Deleting TM1 and replacing it with a cleavable signal sequence or deleting most of the P1 loop, including the SecF binding site, does not inhibit YidC function^40,66^. The auxiliary TM1 and the periplasmic loop are also not present in YidC homologues from Gram-positive bacteria^67^, yet the *B. subtilis* YidC homologs can support SecY-dependent and –independent protein insertion in *E. coli*^68^. Chloroplast Alb3 also lacks TM1 but was still shown to interact with the chloroplast SecY^59^. Our data now show that YidC contains a conserved binding site for SecY within the C1 loop and a second, less-conserved in TM1. The latter appears to be a specific trait in Gram-negative bacteria. Detailed studies on the cooperation of SecYEG with YidC during membrane protein insertion have so far only been performed in *E. coli* and the identification of TM1 as SecY contact site could suggest that YidC-assisted protein insertion via SecYEG in *E. coli* is different from the analogous processes in Gram-positive bacteria or chloroplasts.

The C1 loop of YidC is essential for function, but its exact role is unknown. Deletion of the C1 loop interferes with the insertion of the Pf3 and M13 procoat proteins, but not with the insertion of F_o_c or CyoA^35,69^. We show here that the C1 loop is an important contact site for SecY, SRP and FtsY. We also demonstrate that the C-terminus of YidC, which is not resolved in the X-ray structure^34^, is in contact with SecY (Table 1). Together with previous data demonstrating that the C-terminus of YidC is also in contact with SRP, FtsY and ribosomes^53^ and that the C2 loop and the C-terminus form a composite ribosome binding site^35^, these data support the concept that the cytosolically exposed loops and the C-terminus of YidC constitute a composite docking site for multiple partner proteins. This is similar to the cytosolic loops of SecY, which are in contact with the ribosome, FtsY and SecA^23,57^.

Additional contacts to SecY were observed from YidC’s amphipathic helix EH1, which lies parallel to the plane of the membrane and which connects the P1 loop with TM2^34^. EH1 and the P1 loop were also cross-linked to SecG, supporting the concept that the large periplasmic region of YidC serves as interface for the interaction with SecYEG. Finally, strong contacts to SecY were observed from TM3 and TM4, while cross-links from TM5 were weaker and no cross-links to TM6 were observed. TM3 and TM4 are part of the hydrophilic groove and their vicinity to SecY, which binds to YidC via its lateral gate^29,30^, provides evidence for the concept that substrate TMs are released from the SecY channel into the hydrophilic groove of YidC. TM5 is also part of the hydrophilic groove, but the weaker cross-links to SecY are probably explained by the fact that the selected residues 504 and 505 are located on the more tightly packed periplasmic side of the membrane, which makes these residues less accessible. These multiple contacts between YidC and SecY also explain why the truncation of the C1 loop or the replacement of the TM1 with an unrelated signal sequence does not preclude YidC-SecY contacts.

Although the results of our current and previous cross-linking experiments^29,30^ allow for a deeper insight into the YidC-SecYEG interaction, it is puzzling that SecYEG-YidC cross-links were only observed by immune-detection when SecYEG was overexpressed. This indicates that at the endogenous SecYEG concentrations, only a small number of SecYEG-YidC complexes exist that are not detected by cross-linking and immune-detection. On the other hand, we observed clear cross-links between YidC and the endogenous Ffh, although the cellular Ffh concentration is only half of the SecYEG concentration^1^. Thus, the YidC-SecYEG cross-linking efficiency is limited because most of the endogenous SecYEG complexes are either not accessible to YidC or because SecYEG only transiently associates with YidC during membrane protein insertion. Direct YidC-SecYEG interactions have so far primarily been observed when SecYEG was overproduced^27,43,44^; to our knowledge SecYEG-YidC complexes in native membranes were only detected by Blue-native studies^26,70^, and required the presence of a stalled membrane protein substrate^70^. This is in line with the sequential hand-over of a radioactively-labelled nascent membrane protein from SecY to YidC that was also observed with native membranes^31,32,71^. Thus, in native membranes YidC appears to interact with SecY only during membrane protein insertion. Whether SecYEG translocons not engaged in membrane protein insertion exist just as trimeric SecYEG core or in complex with other Sec translocon interacting proteins^26,72^ is currently unknown. The observation that YidC-SecYEG cross-links are only observed when SecYEG is overexpressed could indicate that under native conditions SecYEG is primarily in contact with partner proteins other than YidC. Only by overexpression sufficient SecYEG is available to form stable complexes with YidC, which otherwise would only transiently exist. The identification of the complete SecYEG interactome will probably reveal further partner proteins and extend the concept of a single holo-translocon^43^ to the presence of variable SecYEG assemblies, which transiently or stably recruit additional partner proteins for facilitating transport of the huge variety of exported proteins in bacteria.

## Methods

### Strains, growth conditions, plasmids and plasmid construction

The following *E. coli* strains and plasmids were used: DH5α^73^, BL21(Merck, Darmstadt, Germany), JS7131^36^, MC4100^74^, pEVol^75^, pBad24-YidC; pBad24-YidC_His_; pTrc99a-SecY_His_EG^57^ and pTrc99a-SecYEG_His_-YidC. Cells were grown in LB medium at either 30 °C or 37 °C; supplemented with 0.2% arabinose for JS7131. For generating a His-tag free SecYEG, the coding sequence for the C-Terminal His-tag in pTrc99a-SecY_His_EG was removed using the Phusion PCR Kit (NE Biolabs, Frankfurt, Germany) with 5′ phosphorylated oligonucleotides SecY_HisR_for and SecY_HisR_rev (Supplementary Table S1), resulting in plasmid pTrc99a-SecYEG. TAG stop codons were incorporated at the indicated positions of pTRc99a-SecY_(His)_EG or pBad24-YidC_His_ using the Phusion PCR Kit or the Pfu Ultra II High Fidelity Polymerase Kit (Agilent, Stratagene, Germany) with 5′-phosphorylated oligonucleotides. pBad24-YidC was constructed by PCR amplification of *yidC* using genomic *E. coli* MC4100 DNA and corresponding oligonucleotide primer (Supplementary Table S1) that inserted *Nco*I and *Xba*1 restriction sites. The PCR product was then cloned via *Nco*I/*Xba*1 into pBad24^76^. Next, the coding sequence for ten consecutive histidine residues, followed by a gly-ser-gly linker were inserted immediately after the ATG start codon of *yidC* by reverse PCR. This resulted in plasmid pBad24-YidC_His_. pTrc99a-SecY_His_EG was used as a template for the generation of pTrc99a-SecY_His_EG-YidC and pTrc99a-SecYEG-YidC_His_ co-expression systems. The His_10_-Gly-Ser-Gly-*yidC* coding sequence insert was amplified from plasmid pBad24-YidC_His_ using primers that contained SalI (5′) and XbaI (3′) restriction sites and the restricted PCR fragment was cloned into pTrc99a-SecY_His_EG. In the resulting pTrc99a-SecY_His_EG-YidC_His_ plasmid, *yidC* contains its own ribosome binding site (AGGAGG) and is located 44 base pairs downstream of the *secG* stop-codon. Subsequently, the His-tag from SecY_His_ was removed using inverse PCR and the primers SecY_HisR_for and SecY_HisR_rev primers which restored the wild type SecY sequence. Alternatively, the His-tag from YidC_His_ was removed using the YidC_HisR_for and YidC_HisR_rev primers.

pTrc99a-SecY_His_EG-YidC was used as a template for the generation of all YidC-pBpa derivatives used in Figs 1b, 2, 3, 4, S1 and S2, as well as for the construction of the SecG deletion used in Fig. 4. pTrc99a-SecYEG-YidC_His_ was used as template for the production of YidC-amber stop codon variants which were used in Figs 1c, 5d and 7. Insertion of the TAG stop codon at position 127 of SecY was made by reverse PCR using the primer SecY_I91_for and SecY_I91_rev with pTrc99a-SecYEG-YidC_His_ as template. The generation of C2 loop deletions Δ3, Δ4, Δ3 + Δ4, the ΔTM1 strain and the point mutation P388A was constructed by reverse PCR using the pTrc99a-SecY(L127TAG)EG-YidC_His_ as template DNA. The pTrc99a-SecYEG-YidC_His_ construct was also used as template for generating the *pelB-YidC* variant. The PelB-YidC-for and PelB-YidC-rev primers (Supplementary Table S1) containing the *pelB* coding sequence were used for replacement of the N-terminal His_10_-Gly-Ser-Gly region and the ATG start codon of His_10_-Gly-Ser-Gly-*yidC* via reverse PCR. Subsequently the ATG start codon was inserted in front of the pelB sequence via reverse PCR using the Rest.pelB-for and Rest.pelB-rev primers (Supplementary Table S1).

### Activity assays for YidC derivatives

The functionality of YidC variants was determined by transforming the indicated plasmids into the conditional YidC-depletion strain JS7131^36^. An overnight LB culture supplemented with 50 μg/μl ampicillin, 50 µg/µl spectinomycin, and 0.2% L-arabinose (for YidC expression) was used to inoculate fresh LB cultures containing 50 μg/μl ampicillin, 50 µg/µl spectinomycin and 0.2% arabinose. These cultures were grown until they reached an OD_600_ = 0.5 and serially diluted with fresh LB medium in multi-well plates. Subsequently, 20 µl of these dilutions were spotted on LB agar plates containing either 0.2% L-arabinose (for YidC expression) or 0.2% fructose (for YidC depletion) and were grown overnight at 37 °C. The functionality of pelB-YidC variant was also assessed by transformation of the pTrc99a_SecYEG-(pelB-YidC) plasmid in the conditional YidC depletion strain JS7131 and overnight growth at 37 °C on LB agar plates containing 50 µg/µl spectinomycin and 0.2% L-arabinose (YidC^+^) or 0.2% Fructose (YidC^−^).

### *In vivo* pBpa cross-linking

BL21 cells containing the pEVol plasmid together with YidC-pBpa variants in pBad24 or pTrc99a plasmids were cultured overnight in LB medium at 37 °C. 10 ml of the overnight culture was further used for inoculation of 1 l LB medium containing 0.5 mM pBpa (in NaOH), 50 μg/μl of ampicillin and 35 μg/μl of chloramphenicol. The cultures were further incubated at 37 °C until they reached the early exponential growth phase (OD_600_ = 0.5–0.8) and induced with 0.02% L-arabinose (for pEVol and pBad24 plasmids) and/or 0.5 mM isopropyl 1-thio-β-D-galactopyranoside (IPTG, for pTrc99a plasmids). After induction, the cultures were grown for 2 hours at 37 °C, cooled down on ice for 10 minutes and harvested by centrifugation at 5000 rpm in a JLA 9.1000 rotor for 10 minutes. The cell pellets were resuspended in 10 ml of PBS buffer (137 mM NaCl, 2.7 mM KCl, 10 mM Na_2_HPO_4_ and 1.76 mM KH_2_PO_4_) and divided in two multi-well plates. One plate was exposed to UV light on ice for 20 minutes (UV chamber: BLX-365, from Vilber Lourmat) while the other plate was kept in the dark. After UV irradiation, the cell suspension was transferred to 50 ml Falcon tubes and cells were collected by centrifugation at 5000 rpm for 10 minutes in a table-top centrifuge. Each cell pellet was resuspended in 10 ml of resuspension buffer (50 mM Tris/HCl pH 7.5, 300 mM NaCl, 10 mM Mg(Ac)_2_) for subsequent YidC purification or 20 mM Tris/HCl pH 7.5, 300 mM NaCl, 5 mM MgCl_2_ for subsequent SecY purification). Next, the samples were lysed by French pressing and the cell debris was removed by centrifugation at 15800 rpm for 30 minutes in a SS34 rotor. The supernatant was further centrifuged at 45000 rpm for 1.5 hours in a TLA 50.2 rotor for membrane sedimentation. Next, the membrane pellets were solubilized in 1% (w/v) n-dodecyl β-D-maltoside (DDM) dissolved in resuspension buffer supplemented with 10% (w/v) glycerol for 1 hour at 4 °C. YidC or SecY were purified via metal affinity chromatography using TALON® Metal affinity resin (Clontech, USA). The samples were incubated for 1 hour at 4 °C with 1 ml/1 l LB culture of pre-equilibrated TALON® Metal affinity resin, washed five times with 10 ml/sample of washing buffer (40 mM Imidazol, 10% glycerol (w/v), 50 mM Tris/HCl, pH 7.5, 300 mM NaCl, 10 mM Mg(Ac)_2_ and 0.03% DDM for YidC purification or 5 mM Imidazol, 10% (w/v) glycerol, 20 mM Tris/HCl pH 7.5, 300 mM NaCl, 5 mM MgCl_2_ and 0.03% DDM for SecY purification) and eluted four times in a total volume of 2 ml elution buffer (200 mM Imidazole, 10% glycerol (w/v), 50 mM Tris/HCl pH 7.5, 300 mM NaCl, 10 mM Mg(Ac)_2_ and 0.03% DDM for YidC purification or 200 mM Imidazole, 10% (w/v) glycerol, 10 ml of 20 mM Tris/HCl pH 7.5, 300 mM NaCl, 5 mM MgCl_2_ and 0.03% DDM for SecY purification). Next the samples were precipitated with 1 volume of 10% trichloracetic acid (TCA), denatured at 56 °C for 10 minutes in 35 μl of TCA loading dye (prepared by mixing one part of Solution III (1 M Dithiothreitol) with 4 parts of Solution II (6.33% SDS (w/v), 0.083 M Tris-Base, 30% glycerol and 0.053% Bromphenol blue) and 5 parts of Solution I (0.2 M Tris, 0.02 M EDTA pH 8) and analysed on SDS Page by western blotting or mass-spectrometry.

### *In vivo* para-formaldehyde cross-linking

BL21 cells carrying the pBad24-YidC_His_ or the pTrc99a-SecYEG-YidC_His_ plasmids were used for inoculation of two flasks containing 1 l of LB medium with 50 µg/ml ampicillin. Both cultures were grown at 37 °C until they reached early exponential growth phase (OD_600_ = 0.5–0.8) and were then induced with 0.02% L-arabinose (pBad24-YidC_His_) or 0.5 mM IPTG (pTrc99a-SecYEG-YidC_His_). The cells were further grown at 37 °C for 2 hours to allow expression. Next, 50 ml of freshly dissolved para-formaldehyde (PFA) in para-formaldehyde buffer (PFA buffer) (2.7 mM KCl, 1.8 mM KH_2_PO_4_, 10 mM Na_2_HPO_4_, pH 6.5, final PFA concentration 1 g PFA/50 ml PFA buffer) was added to one flask, while only PFA buffer was added to the other flask. Both samples were further incubated at 37 °C for 30 minutes, cooled down on ice for 10 minutes and harvested by centrifugation in a JLA 9.1000 rotor at 5.000 rpm for 10 minutes. Subsequently the cell pellets were dissolved in 10 ml of resuspension buffer (50 mM Tris/HCl pH 7.5, 300 mM NaCl, 10 mM Mg(Ac)_2_) and lysed by French pressing followed by clearance of cell debris at 15.800 rpm for 30 min in a SS34 rotor. Next, the supernatant was used to sediment the membranes by centrifugation at 45.000 rpm for 1.5 hours in a TLA 50.2 rotor. The membranes were further solubilized in 1% DDM dissolved in resuspension buffer supplemented with 10% glycerol. Next, YidC was purified via metal affinity chromatography using TALON® Metal affinity resin as described above and analyzed on SDS Page by western blotting. In order to preserve the PFA cross-links, which are temperature sensitive, the samples were denatured in TCA loading dye at 37 °C for 18 minutes.

### Preparation of material for mass spectrometric analyses

*E. coli* BL21 cells carrying plasmid pEVol and plasmid pTrc99a-SecY_His_EG were cultured overnight at 37 °C in LB medium containing 50 µg/ml ampicillin and 30 µg/ml chloramphenicol. 10 ml of the overnight culture was further used for inoculation in 1 l minimal medium (1% w/v glycerol, 47.7 mM Na_2_HPO_4_, 22 mM KH_2_PO_4_, 8.6 mM NaCl, 18.7 mM NH_4_Cl, 0.153 mM CaCl_2_, 0.59 mM MgSO_4_, 2.07 µM MoNa_2_O_4_, 4.198 µM CoSO_4_, 37.49 µM MnSO_4_, 4.35 µM ZnSO_4_, 6.29 µM FeCl_2_, 16.17 µM H_3_BO_3_, 40 mg/l L-leucine, 1 mg/ml D-biotin, 1 mg/ml thiamine, 7 mg/l chloramphenicol, 10 mg/l ampicillin) until cells reached the early exponential growth phase (OD_600_ = 0.5–0.8) and protein expression was then induced with 0.5 mM IPTG. After induction, the cultures were grown for 4 hours at 30 °C, were cooled down on ice for 10 minutes and harvested by centrifugation at 5.000 rpm in a JLA 9.1000 rotor for 10 minutes. Each cell pellet was resuspended in 10 ml resuspension buffer (20 mM HEPES, pH 7.5, 300 mM NaCl, 5 mM MgCl_2_) to which 0.5 mM PMSF and 2 pills/l cOmplete™ protease inhibitor cocktail (Roche®; Mannheim Germany) were added. Next, the samples were lysed by French pressing and cell debris was removed by centrifugation at 15.800 rpm for 30 minutes in an SS34 rotor. The supernatant was further centrifuged at 45.000 rpm for 1.5 hours in a TLA 50.2 rotor for membrane sedimentation. Next, the membrane pellets were solubilized in solubilisation buffer (20 mM HEPES, pH 7.5, 300 mM NaCl, 5 mM MgCl_2_, 10% glycerol, 1% DDM) for 1 hour at 4 °C. Non-solubilized membranes were separated by centrifugation for 10 minutes at 14.000 rpm at 4 °C. The supernatant was transferred onto TALON® Metal affinity resin, which was then washed twice with washing buffer (20 mM HEPES pH 7.5, 300 mM NaCl, 5 mM MgCl_2_, 10% glycerol, 0.03% DDM, 5 mM imidazole) and subsequently washed four times with imidazole-free washing buffer. Further, the material was washed four times with imidazole-containing washing buffer and eluted in 2 ml elution buffer (20 mM HEPES pH 7.5, 300 mM NaCl, 5 mM MgCl_2_, 10% glycerol, 0.03% DDM, 200 mM imidazole).

The chemical cross-linker disuccinimidyl suberate (DSS, ThermoFisher Scientific, Langenselbold, Germany) was dissolved in DMSO and incubated with the samples for 30 minutes at 25 °C (final concentration DSS: 1 mM) after the second-imidazole-free washing step (on-column cross-linking) or after the elution step (solution cross-linking). After the DSS incubation step, the reaction was quenched by adding Tris/HCl, pH 7.5 to a final concentration of 20 mM.

Protein samples for mass spectrometry analyses were precipitated with acetone at −20 °C over-night and subsequently centrifuged for 20 minutes at 4 °C and 14.000 rpm. After the supernatant was removed, the protein samples were dried and denatured in 10 µl 8 M urea (in 50 mM NH_4_HCO_3_)_._ Then, 1.1 µl 50 mM Tris(2-carboxyethyl)phosphine (in 50 mM NH_4_HCO_3_) was added and the sample was incubated for 20 minutes at 37 °C with continuous shaking at 600 rpm. Subsequently, 1.23 µl 500 mM iodoacetamide (in 50 mM NH_4_HCO_3_) was added and incubated for 20 minutes in the absence of light. Finally, 3 µl of 100 mM dithiothreitol (in 50 mM NH_4_HCO_3_) and subsequently 25 µl of 50 mM NH_4_HCO_3_ were added. The protein samples were digested overnight with 0.125 µg/µl trypsin at 37 °C, and then acidified with 0.1% trifluoroacetic acid and centrifuged for 6 minutes at 12.000 rpm.

Peptide mixtures were analyzed by liquid chromatography-tandem mass spectrometry (LC-MS/MS) using an Ultimate 3000 RSLCnano coupled to a Q Exactive Plus (Thermo Fisher Scientific) mass spectrometer as described^77^. Proteins were identified using the MaxQuant software package (version 1.5.4.0,^78^) searching against the UniProt *E. coli* proteome set sequence database (version 2016_03). Identification of cross-linked peptides was performed essentially as described^77^ using the program pLink®^79^ for searching inter- or intra-molecular cross-links generated by amine-specific cross-linker DSS between peptides from SecY, SecE, SecG and YidC. The resulting set of peptide spectrum matches at a false discovery rate <0.05 was further filtered requiring a minimum E-value of 0.05 and identification of a pair of cross-linked residues in at least two independent experiments.

### Reproducibility statement

All experiments shown in this study were performed at least twice as independent biological replicates. Each replicate consisted of at least two technical replicates. Only representative western blots and agar plates are displayed. Uncropped images of all western blots and LB plates are provided in the supplementary information files (Supplementary Figure S4).

### Data availability statement

All data generated or analysed during this study are included in this published article (and its Supplementary Information files) or are available from the corresponding author on reasonable request.

## Electronic supplementary material

Supplementary Information
