# Supplementary material for: The interaction network of the YidC insertase with the SecYEG translocon, SRP and the SRP receptor FtsY
Source: Sci Rep. 2018 Jan 12;8:578. doi: 10.1038/s41598-017-19019-w (PMC5766551; doi:10.1038/s41598-017-19019-w)
Supplement: Supplementary file 1 — Supplementary Information [file 41598_2017_19019_MOESM1_ESM.pdf]

Supplementary information to

The interaction network of the YidC insertase with the SecYEG translocon, SRP and the SRP receptor FtsY

Narcis-Adrian Petriman[1,2], Benjamin Jauß[1], Antonia Hufnagel[1], Lisa Franz[1], Ilie Sachelaru[1,2], Friedel Drepper[3,4], Bettina Warscheid[3,4] and Hans-Georg Koch[1,*]

[1]Institute of Biochemistry and Molecular Biology, ZBMZ, Faculty of Medicine, Albert-Ludwigs-University Freiburg, 79104 Freiburg Germany, [2]Faculty of Biology, Albert-Ludwigs-University Freiburg, 79104 Freiburg, Germany, [3]Institute of Biology II, Biochemistry – Functional Proteomics, Faculty of Biology, Albert-Ludwigs-University Freiburg, 79104 Freiburg, Germany, [4]BIOSS Centre for Biological Signalling Studies, Albert-Ludwigs-University Freiburg, 79104 Freiburg, Germany

Running title: YidC-SecYEG interaction

*Correspondence should be addressed to: Hans-Georg Koch, Institute of Biochemistry and Molecular Biology, ZBMZ, Faculty of Medicine, Albert-Ludwigs-University Freiburg, Stefan-Meier Strasse 17; 79104 Freiburg, Germany, Telephone 0049-761-2035250; Fax: 0049-761-203-5289; E-mail: Hans-Georg.Koch@biochemie.uni-freiburg.de

Supplementary Figure legends

Supplementary Figure S1: YidC makes multiple contacts to SecY. The *in vivo* photo-cross-linking material produced for figure **2a, 2b and 2c** was analysed on western blot with α-SecY antibodies. Similarly the material produced for figure **2d** was analysed with α-SecY antibodies. Indicated are the UV dependent 95kDa YidC-SecY cross-links as revealed by antibodies directed against SecY (**a-d**). The material was purified via a His-tag on SecY before immune detection. 2xSecY probably corresponds to a SecY dimer that is not dissociated due to the denaturation at 37 °C. Note, that due to the weak signal in (**a, c & d**), a long exposure time was required.

Supplementary Figure S2: YidC makes multiple contacts to SecE and SecG. *In vivo* photo-cross-linking was performed with the co-expression system using YidC variants which contained pBpa at the indicated positions. Cross-linked material was subsequently purified via the His-tag on SecY. The analysis of the purified material on western blot with SecE antibodies (**a** and **b**) or SecG antibodies (**c**) revealed YidC-SecE and YidC-SecG cross-links migrating at about 65kDa. * indicates a possible proteolysis product of the YidC-SecG cross-link.

Supplementary Figure S3: TM1 is not required for the interaction with SecY. (a) Expression test for the Δ2-23YidC variant. 1 x 10^8 cells were collected before (-) and after (+) addition of IPTG, precipitated with 5% trichloroacetic acid (TCA) and after western transfer decorated with α-YidC (upper panel) and α-His antibodies (lower panel). (**b**) A pelB-YidC construct in which TM1 was replaced by the cleavable PelB signal sequence was tested in the co-expression system for its complementation of the conditional *yidC* deletion strain JS7131 as described in the legend to Figure 5. As a control the plasmid-free JS7131 strain (empty) and a JS7131 strain just expressing SecYEG were tested.

Supplementary Figure S4: Uncropped images of all western blots and LB plates used in this study. Labelling of the panels corresponds to the figure labeling in the manuscript and the supplementary information. The numbers in the right margin of each panel corresponds to the molecular weight marker.

Supplementary Table S1: Oligonucleotides used in this study

Primer's name	Sequence (5' to 3')	Template DNA	Purpose of use
For_YidC_NcoI	cggaccatttgataccatggagaacactaacgatgg	*E. coli* MC4100 genomic DNA	Amplification of NcoI_YidC_XbaI DNA fragment
Rev_YidC_XbaI	gaaaaaaggcggtcaatctagaccgccattattttagcg		
For_10XH_YidC_pBad	atcaccatcacggcagcggagattcgcaacgcaatctt ttag	pBad24-YidC	$(his)_{10}$-gly-ser-gly- pBad24-YidC
Rev_10XH_YidC_pBad	ggtgatggtgatggtgatgcatcgttagtgttctccatgg	pBad24-YidC	$(his)_{10}$-gly-ser-gly- pBad24-YidC
D399_YidC_for	cgtctgggctaggacaaacagcg	YidC gene	D399pBpa YidC
R396_YidC_rev	ctcacgcattgcctgaatcttcg	YidC gene	
D236pBpa_for	tagaccattgccgataacg	YidC gene	D236pBpa YidC
D236pBpa_rev	gaacttgtatttctcatac	YidC gene	
F14pBpa_for	taggtgtctttcatgatctg	YidC gene	F14pBpa YidC
F14pBpa_rev	cagcaaagcgatgactaaaag	YidC gene	
V15pBpa_for	tagtctttcatgatctggc	YidC gene	V15pBpa YidC
V15pBpa_rev	gaacagcaaagcgatgact	YidC gene	
L326pBpa_for	tagaccgttgattacggttgg	YidC gene	L326pBpa YidC
L326pBpa_rev	atccaggtgcggagcaac	YidC gene	
F335_YidC_for	tagatctctcagccgctgttcaaac	YidC gene	F335pBpa YidC
F335_YidC_rev	ccaaaaccaaccgtaatcaacggtc	YidC gene	
F350_YidC_for	taggtgggtaactggggcttctcc	YidC gene	F350pBpa YidC
F350_YidC_rev	gctatggatccatttcagcagtttg	YidC gene	
YidC_P431pBpa_for	tagatcttcctggcgttgtac	YidC gene	P431pBpa YidC
YidC_P431pBpa_rev	catctggatcagcagcggg	YidC gene	
V473_YidC_for	atcctgatgggctagacgatgttc	YidC gene	V473pBpa YidC
V500_YidC_rev	cggcataaaggtcatgatcttctg	YidC gene	
V500_YidC_for	tagatcttcaccgtgttcttcctg	YidC gene	V500pBpa YidC
V500_YidC_rev	cggcataaaggtcatgatcttctg	YidC gene	
V504_YidC_for	gtcatcttcacctagttcttcctg	YidC gene	V504pBpa YidC
V500_YidC_rev	cggcataaaggtcatgatcttctg	YidC gene	
F505YidC_for	tagttcctgtggttcccgtc	YidC gene	F505pBpa YidC
F505YidC_rev	cacggtgaagatgaccggca	YidC gene	
Δ3YidC_for	aaacagcgtatcagccagg	YidC gene	392-401 YidC deletion
Δ3YidC_rev	tgcctgaatcttcggctgcaa	YidC gene	
Δ4YidC_for	gcgctgtacaaagctgaga	YidC gene	401-409 YidC deletion
Δ4YidC_rev	tttgtcatcgcccagacgc	YidC gene	
P388A_for	gcgaagattcaggcaatgcgtg	YidC gene	Re-orientation of CH_1-CH_2 loop of YidC
P388A_rev	ctgcaacatacgcatcttcgc	YidC gene	
deltaSecG_for	tctagagggctagcaggag	pTrc99a-$SecY_{His}EG$-V15pBpa YidC and pTrc99a $SecY_{His}EG$	SecG deletion
deltaSecG_rev	aatcaactcctggatcctt		

		-D236pBpa YidC	
SecY-L127-for (60)	gtgtaggcaatattccagtcgatc	pTrc99a-SecYEG-YidC$_{His}$	pTrc99a-L127pBpa SecYEG-YidC$_{His}$
SecY-L127-rev (60)	cagagtaccgtagcgggtgtactg		
YidC_cutpBadl_for	cgcgatctagagggctagcaggaggaattcac	pBad24-YidC$_{His}$	Amplification of shine dalgarno sequence and *yidC$_{His}$*
YidC_cutpBadl_rev	cggtcgtcgactcaggatttttcttctcgcg		
SecY_HisR_for	tactagggccgataaatcgatggaggttttaattcatg	pTrc99a-SecYEG-YidC$_{His}$ and pTrc99a-SecY$_{His}$EG-YidC	removing His-Tag from SecY$_{His}$EG and restoring of wild type SecY sequence
SecY_HisR_rev	gcctttcaggttcgccttcttcaatgc		
YidC_HisR_for	ggcagcggagattcgc	pTrc99a-SecY$_{His}$EG-YidC$_{His}$	removing His-Tag from YidC$_{His}$
YidC_HisR_rev	catcgttagtgttctccatggtg		
V519pBa-for	agcaacctggtaaccatta	pTrc99a-SecY$_{His}$EG-YidC	V519pBpa YidC
V519pBpa-rev	ctagatatagtacagcaccag		
L513 YidC_for	taggtgctgtactatatc	pTrc99a-SecY$_{His}$EG-YidC	V519pBpa YidC
L513 YidC_rev	acctgacgggaaccacagg		
YidC K249-forw	tagggtggttgggtggcgatgctgc	pTrc99a-SecY$_{His}$EG-YidC	K249pBpa YidC
YidC K249-rev	cgaagagatgttcaggttttcgttatcg		
PelB-YidC-for	tgctgctcctcgctgcccagccggcgatggccgggaa actgatctcggttaag	pTrc99a-SecYEG-YidC$_{His}$	Replacement of the 1-56 amino acid region of YidC with pelB signal sequence
PelB-YidC-rev	gaccagcagcagcggtcggcagcaggtatttcgttagt gttctccatggtga		
Rest.pelB-for	aaatacctgctgccgaccgaccg	pTrc99a-SecYEG-(pelB-YidC)	Addition of ATG in front of the pelB signal sequence
Rest.pelB-rev	catcgttagtgttctccatggtg		

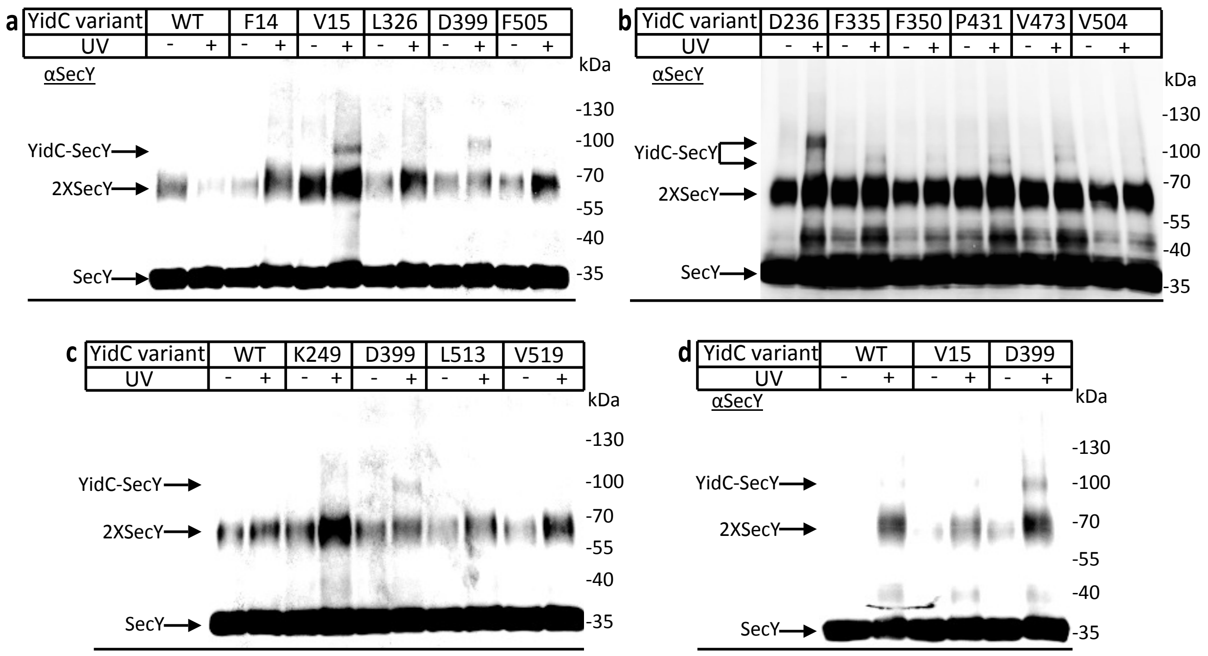

(Petriman et al., Fig. S1)

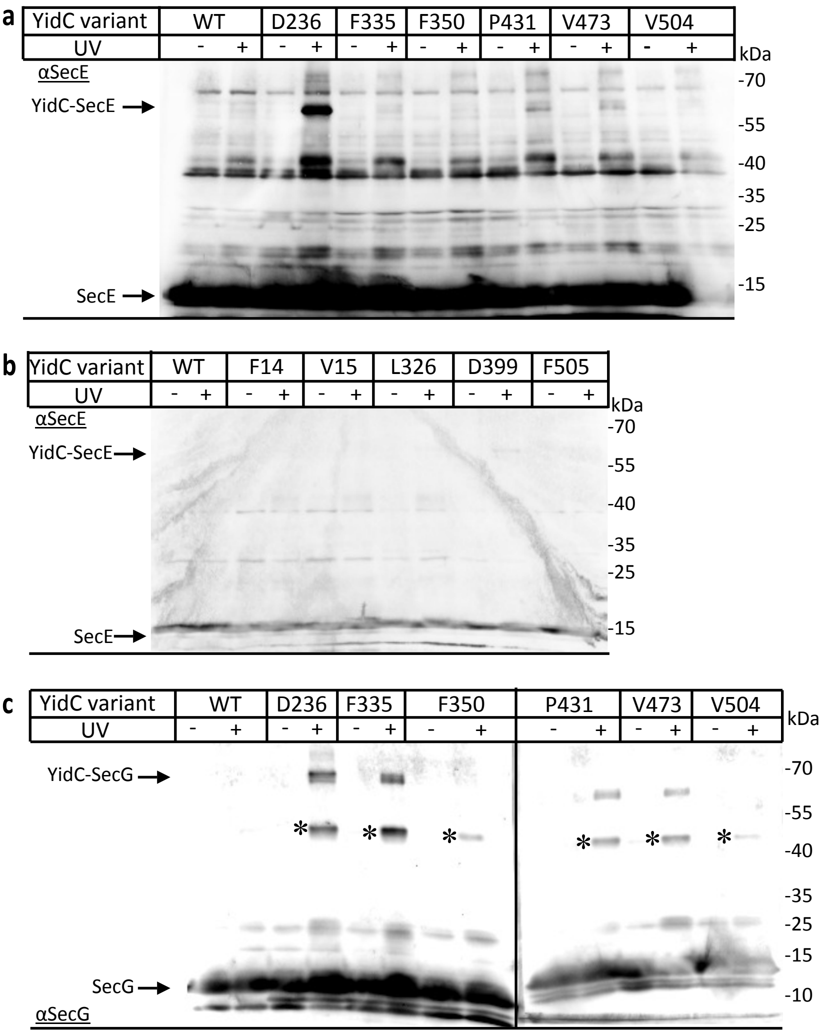

(Petriman et al., Fig. S2)

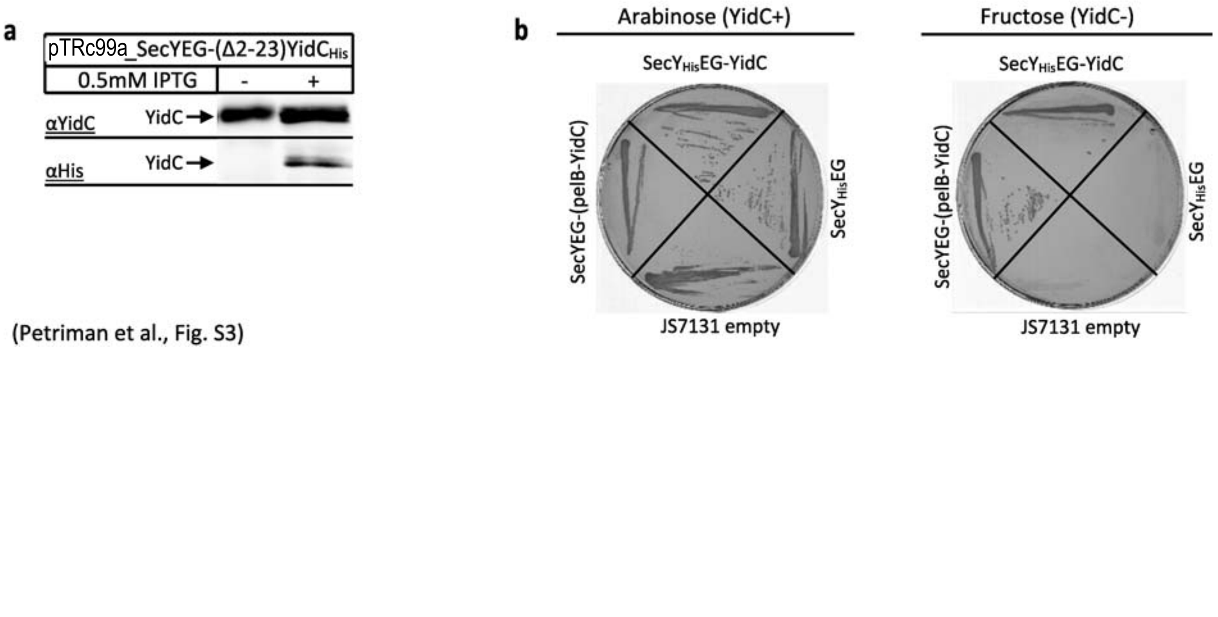

(Petriman et al., Fig. S3)

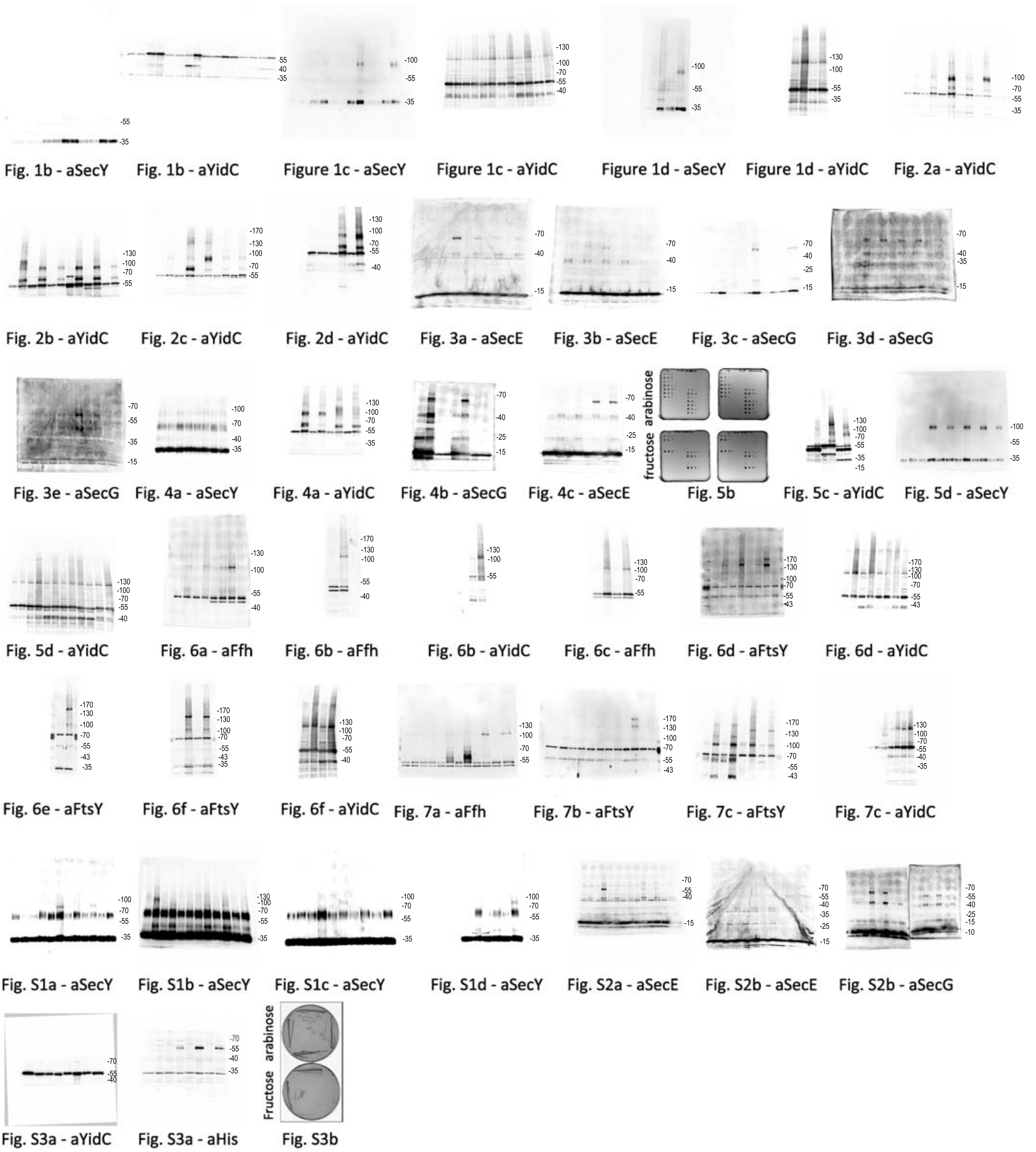

(Petriman et al., Fig. S4)
